# C-type lectin receptors Mcl and Mincle control development of multiple sclerosis–like neuroinflammation

**DOI:** 10.1172/JCI125857

**Published:** 2020-01-13

**Authors:** Marie N’diaye, Susanna Brauner, Sevasti Flytzani, Lara Kular, Andreas Warnecke, Milena Z. Adzemovic, Eliane Piket, Jin-Hong Min, Will Edwards, Filia Mela, Hoi Ying Choi, Vera Magg, Tojo James, Magdalena Linden, Holger M. Reichardt, Michael R. Daws, Jack van Horssen, Ingrid Kockum, Robert A. Harris, Tomas Olsson, Andre O. Guerreiro-Cacais, Maja Jagodic

**Affiliations:** 1 Department of Clinical Neuroscience, Center for Molecular Medicine, Karolinska Institutet, Stockholm, Sweden.; 2 Institute for Cellular and Molecular Immunology, University Medical Center Göttingen, Göttingen, Germany.; 3 Department of Anatomy, University of Oslo, Oslo, Norway.; 4 Department of Molecular Cell Biology and Immunology, Amsterdam Neuroscience, MS Center Amsterdam, Amsterdam University Medical Center, Amsterdam, Netherlands.

**Keywords:** Autoimmunity, Immunology, Antigen presenting cells, Innate immunity, Multiple sclerosis

## Abstract

Pattern recognition receptors (PRRs) are crucial for responses to infections and tissue damage; however, their role in autoimmunity is less clear. Herein we demonstrate that 2 C-type lectin receptors (CLRs) Mcl and Mincle play an important role in the pathogenesis of experimental autoimmune encephalomyelitis (EAE), an animal model of multiple sclerosis (MS). Congenic rats expressing lower levels of Mcl and Mincle on myeloid cells exhibited a drastic reduction in EAE incidence. In vivo silencing of *Mcl* and *Mincle* or blockade of their endogenous ligand SAP130 revealed that these receptors’ expression in the central nervous system is crucial for T cell recruitment and reactivation into a pathogenic Th17/GM-CSF phenotype. Consistent with this, we uncovered MCL- and MINCLE-expressing cells in brain lesions of MS patients and we further found an upregulation of the MCL/MINCLE signaling pathway and an increased response following MCL/MINCLE stimulation in peripheral blood mononuclear cells from MS patients. Together, these data support a role for CLRs in autoimmunity and implicate the MCL/MINCLE pathway as a potential therapeutic target in MS.

## Graphical abstract

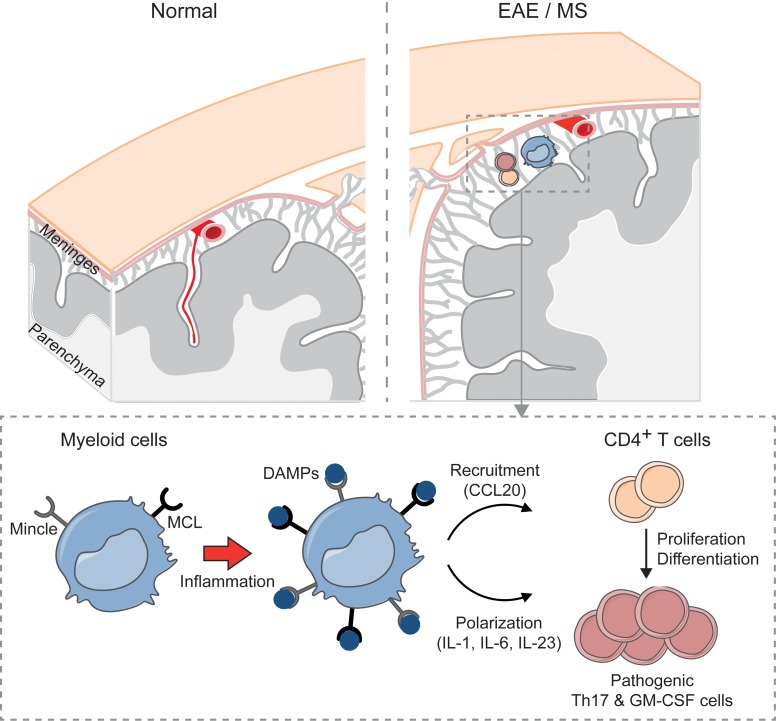

## Introduction

Multiple sclerosis (MS), a leading cause of neurological disability in young adults, is a chronic inflammatory disease of the central nervous system (CNS) characterized by autoimmune destruction of myelin and subsequent neuroaxonal loss. Autoreactive CD4^+^ T cells infiltrating the CNS are considered to be important triggers of MS pathogenesis (1). Antigen-presenting cells (APCs) play a crucial role in the reactivation of CD4^+^ T cells, which drive the subsequent pathogenic inflammatory responses (2–4). However, the mechanisms by which T cell fate is determined upon reactivation in the CNS remain poorly understood. A growing body of evidence suggests a role for pattern recognition receptors (PRRs) in autoimmune inflammatory responses (5). Previous studies have primarily focused on Toll-like receptors (TLRs), showing their potential role in modulating pathogenic disease pathways (6–8); however, little is known about the role of C-type lectin receptors (CLRs) in autoimmunity and particularly in MS (9).

Myeloid CLRs are important sensors of infectious agents and tissue homeostasis through recognition of pathogen-associated molecular patterns (PAMPs) and damage-associated molecular patterns (DAMPs) (10, 11). In addition to their well-known role in infections and tissue damage, recent animal studies have implicated several CLRs as risk genes for autoimmune diseases including arthritis and experimental autoimmune encephalomyelitis (EAE), an MS-like disease model (12, 13). Among the CLRs, CLEC4D (MCL) and CLEC4E (MINCLE), 2 members of the Dectin-2 family primarily coexpressed on myeloid cells (14), have recently gained attention in human inflammatory diseases. MCL and MINCLE signaling leads to the activation of NF-κB via the BCL10/CARD9/MALT1 pathway (11, 15). Interestingly, several members of this pathway have been identified as candidate risk genes for multiple chronic inflammatory diseases such as Crohn’s disease, ankylosing spondylitis, ulcerative colitis, inflammatory bowel disease, and MS (16, 17).

In the present study, we investigated the role of Mcl and Mincle in EAE and MS. Myelin oligodendrocyte glycoprotein–induced (MOG-induced) EAE in rats provides a robust model of chronic relapsing-remitting disease, recapitulating several clinical and histopathological features of MS (18). We exploited the fact that the Dark Agouti (DA) strain is highly susceptible to EAE, while the Piebald Virol Glaxo (PVG) strain is relatively resistant (18), and thus generated a congenic strain containing a PVG region encompassing CLR genes on the DA background. Herein we report that this strain exhibits reduced expression of Mcl and Mincle on myeloid cells and is resistant to EAE induction. Mechanistically, we demonstrate that meningeal myeloid cells orchestrate the recruitment of T cells to the CNS and their reactivation into a pathogenic Th17/GM-CSF phenotype via the Mcl/Mincle pathway. This mechanism is likely triggered, at least in part, by the alarmin SAP130 during early disease development. Both MCL and MINCLE are highly expressed on infiltrating macrophages in active MS lesions. Notably, we show that the MCL/MINCLE pathway is affected in MS, with high expression of the receptors on immune cells associating with both increased MS disease activity and progression, and that MCL/MINCLE stimulation ex vivo results in higher cytokine production in peripheral blood mononuclear cells (PBMCs) of MS patients compared with healthy controls.

## Results

### CLR gene expression in immune cells modulates EAE development.

**Figure 1:**
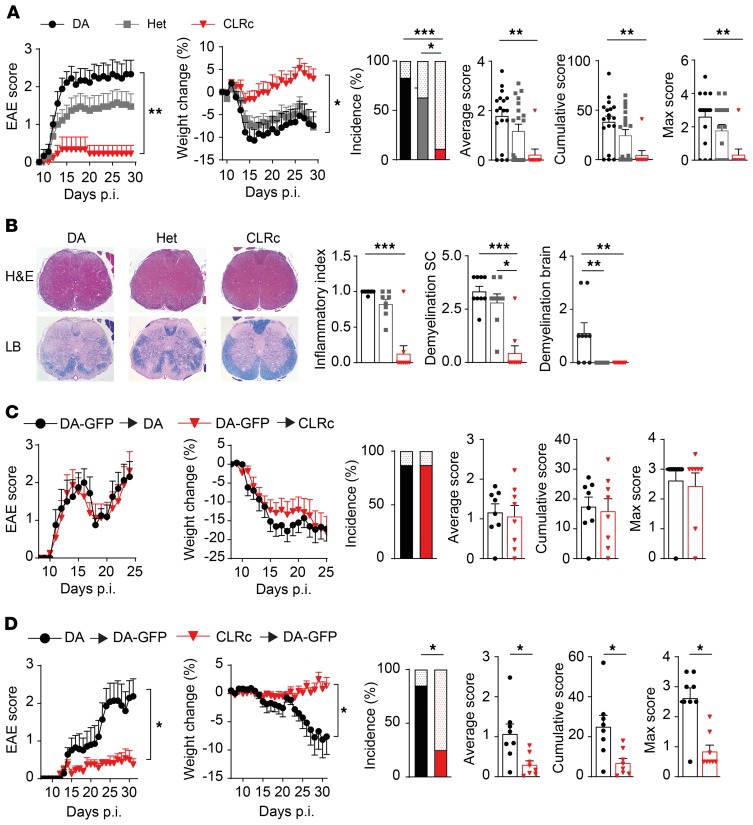
CLRc rats are protected from EAE in a peripheral immune cell–dependent manner. Homozygous (CLRc) and heterozygous (Het) congenic rats and their littermate DA controls were immunized with MOG and followed for signs of disease. (**A**) Clinical signs of EAE and disease parameters in DA littermate controls (*n* = 18), Het (*n* = 19), and CLRc rats (*n* = 9) (representative of 3 experiments). For EAE incidence, the upper dotted bars represent unaffected rats, whereas the lower plain bars represent affected rats. (**B**) Histopathological analysis of spinal cord (SC) on day 29. Left: Representative images of H&E and Luxol fast blue (LB) staining (original magnification, ×40). Right: Quantification of inflammation and demyelination for DA (*n* = 9), Het (*n* = 8), and CLRc rats (*n* = 9). (**C** and **D**) Lethally irradiated rats were transplanted with bone marrow (BM) from donor animals, reconstituted for 2 months, and then immunized with MOG. Clinical signs of EAE and disease parameters were assessed in (**C**) DA or CLRc recipient rats transplanted with DA-GFP BM (DA-GFP → DA [*n* = 8] or DA-GFP → CLRc [*n* = 8]) and (**D**) DA-GFP recipients transplanted with DA or CLRc BM (CLRc → DA-GFP [*n* = 8] and DA → DA-GFP [*n* = 7]). Data are presented as the mean ± SEM. The following statistical tests were used: 1-way ANOVA with Dunnett’s multiple-comparisons test (**A**, for area under the curve [AUC] of clinical EAE and weight change), Kruskal-Wallis test with Dunn’s multiple-comparisons test (**A** [for average, cumulative, and max EAE score] and **B**), unpaired 2-tailed *t* test (**C** and **D**, for AUC of clinical EAE and weight change), Mann-Whitney *U* test (**C** and **D**, for average, cumulative, and max EAE score), and χ^2^ test (**A**, **C**, and **D**, for EAE incidence). *P < 0.05; **P < 0.01; ***P < 0.001.

We have previously shown that a genetic region on rat chromosome 4 encompassing a cluster of CLRs confers resistance to EAE when transferred from the resistant PVG strain to the susceptible DA strain (12, 19). In order to identify the gene(s) responsible for this protective effect, we have further reduced the size of the transferred region in a congenic strain (hereafter referred to as CLRc) comprising 5 genes, 4 of which belong to the Dectin-2 family (Supplemental Table 1; supplemental material available online with this article; https://doi.org/10.1172/JCI125857DS1). Upon MOG immunization, the homozygous CLRc rats displayed marked protection compared with DA homozygous littermate controls, while heterozygous rats exhibited an intermediate phenotype (Figure 1A). In accordance, weight loss in CLRc was significantly milder, recovery was faster, and disease incidence as well as average, cumulative, and max scores were lower compared with DA rats (Figure 1A). Histopathological investigation of the spinal cord on day 29 postimmunization (p.i.) revealed significant reduction in both extent of demyelination and inflammatory index in CLRc rats (Figure 1B and Supplemental Figure 1A). Interestingly, only DA rats presented demyelination in the brain, while this was not observed in either CLRc or heterozygous rats (Figure 1B and Supplemental Figure 1B). These results indicate that the PVG alleles encompassing the CLR genes confer resistance to EAE.

We next examined the contribution of CLRs in CNS-derived versus bone marrow–derived (BM-derived) immune cells using BM-chimeric rats. Reconstitution of lethally irradiated DA or CLRc rats with BM isolated from DA-GFP rats led to the replacement of peripheral immune cells, as well as meningeal and perivascular macrophages with donor BM (Supplemental Figure 1, C–F). No significant differences in EAE development, weight change, incidence, or average cumulative and max score were observed between the strains (Figure 1C). However, the transplantation of DA-GFP rats with DA or CLRc BM recapitulated the phenotype observed in congenic animals, with CLRc → DA-GFP chimeric rats displaying significantly reduced disease incidence and weight and reduced average, cumulative, and max scores compared with DA → DA-GFP chimeras (Figure 1D and Supplemental Figure 1, G and H).

These findings suggest that the protective effect of the CLRc is mediated through the peripheral immune cell compartment and/or the meningeal/perivascular macrophages and not via the CNS-resident microglia.

### The CLRc locus modulates activation of peripheral T cells recruited to the CNS.

**Figure 2:**
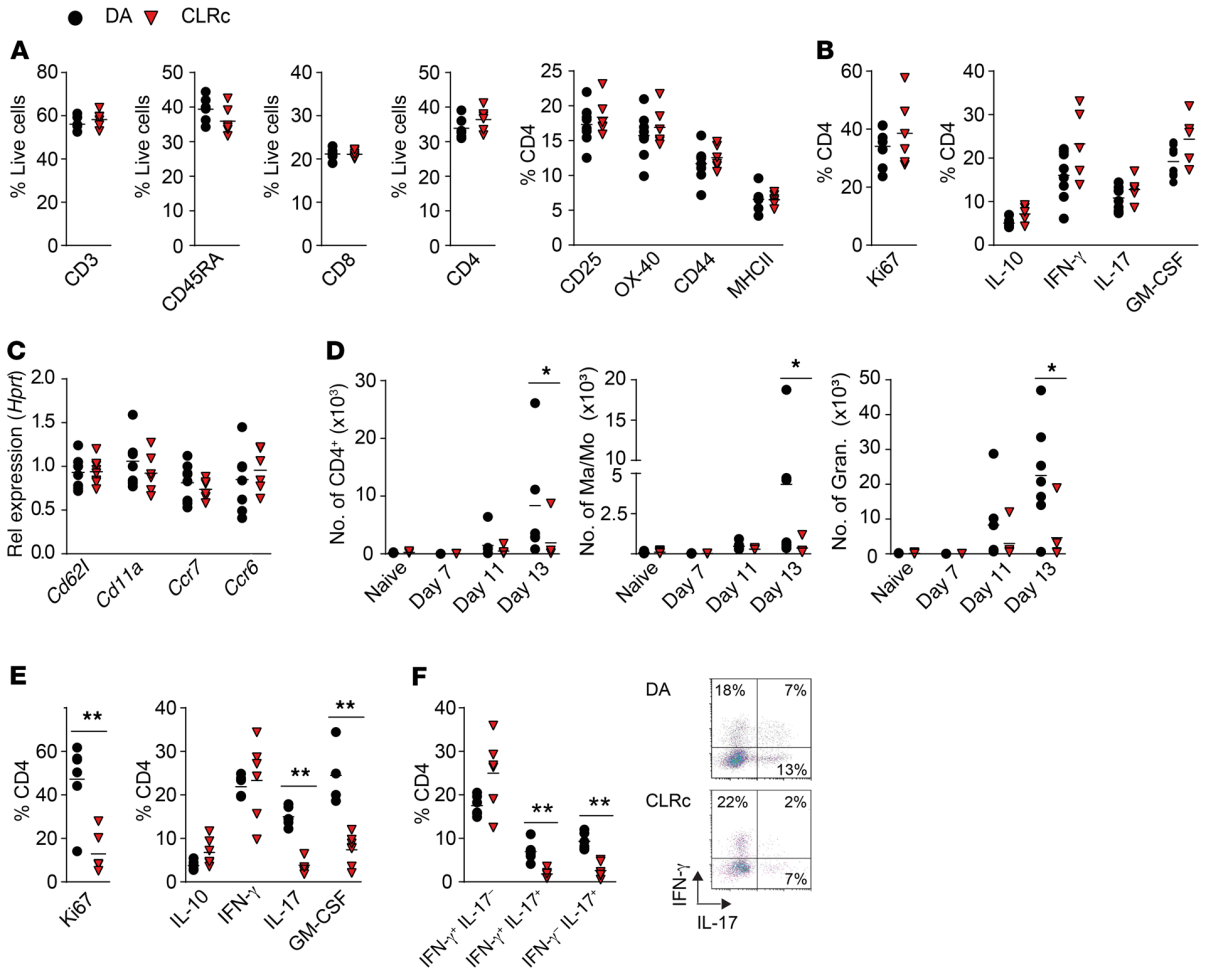
Modulation of T cell activation in the CNS, but not in the periphery, underlies the protective CLRc phenotype. Homozygous CLRc rats and littermate DA controls were immunized with MOG and phenotyped during the disease course. Characterization of cells infiltrating draining lymph nodes (dLNs) and the CNS. (**A**) Frequency of total T cells (CD3), B cells (CD45RA), and CD8^+^ and CD4^+^ T cells on day 7 p.i. in dLNs of DA (*n* = 6–8) and CLRc (*n* = 6) rats. Frequency of activation markers on CD4^+^ T cells (representative of 4 experiments). (**B**) Ki67 expression and cytokine production on day 7 p.i. in CD4^+^ T cells from dLNs of DA (*n* = 7–8) and CLRc (*n* = 5–6) rats following 72-hour in vitro MOG stimulation (representative of 3 experiments). (**C**) qPCR for *Cd62l*, *Cd11a*, *Ccr7*, and *Ccr6* in CD4^+^ T cells sorted from dLNs of DA (*n* = 6) and CLRc (*n* = 6) rats on day 7 p.i. (representative of 2 experiments). (**D**) Number of infiltrating CD4^+^ T cells, macrophages/monocytes (Ma/Mo), and granulocytes in spinal cord of naive DA and CLRc rats (*n* = 12 and *n* = 12) and at 7 (*n* = 3 and *n* = 3), 11 (*n* = 6 and *n* = 5), and 13 days p.i. (*n* = 7 and *n* = 5) (representative of 2 experiments). (**E** and **F**) Characterization of proliferation and cytokine production in infiltrating cells isolated from spinal cord on day 13 p.i. stimulated in vitro with PMA/ionomycin/brefeldin A for 5 hours in CLRc rats (*n* = 6) and DA rats (*n* = 6) (representative of 2 experiments). Data are presented as the mean ± SEM. All comparisons were analyzed with the Mann-Whitney *U* test. *P < 0.05; **P < 0.01.

Myeloid APCs play a pivotal role in T cell activation and subsequent EAE progression (2–4). Because C-type lectins are primarily expressed on myeloid cells (20) we first investigated whether the lower EAE incidence in CLRc rats resulted from an incomplete activation of T cells during the priming phase. Characterization of draining lymph nodes on day 7 p.i. revealed no differences in CD4^+^ T cell infiltration, activation, cytokine production, or proliferation, implying that peripheral priming is unaffected in CLRc rats (Figure 2, A–C, and Supplemental Figure 2, A and B). However, analysis of cellular infiltration in the spinal cord at disease onset (day 13 p.i.) revealed reduced numbers of CD4^+^ T cells, monocytes/macrophages, and granulocytes in CLRc compared with DA rats (Figure 2D and Supplemental Figure 2C). Further characterization of the infiltrating CD4^+^ T cells revealed a significant reduction of Ki67^+^ cells, a marker of proliferation (Figure 2E), along with reduced proportions of IL-17–producing, IL-17/IFN-γ double-producing, and GM-CSF–producing T cells (Figure 2, E and F), regarded as the most inflammatory T cell phenotypes in the context of EAE (21–26). No significant differences were observed in IL-10 and IFN-γ production (Figure 2, E and F). Evaluation of meninges and the spinal cord prior to clinical signs of EAE revealed a 2- to 4-fold more extensive infiltration of macrophages, monocytes, granulocytes, and CD4^+^ T cells in the meninges compared with the spinal cord (Supplemental Figure 1I), regardless of the genotype. This finding strengthens the notion that the most likely site for reactivation of antigen-specific T cells is in the meninges, as previously suggested (3, 4).

Overall, these results suggest that CLRs modulate the reactivation of T cells within the CNS, most likely in the meninges, and participate in the differentiation or maintenance of IL-17– and GM-CSF–producing cells.

### Reduced expression of Mcl and Mincle in monocytes/macrophages associates with protection from EAE.

**Figure 3:**
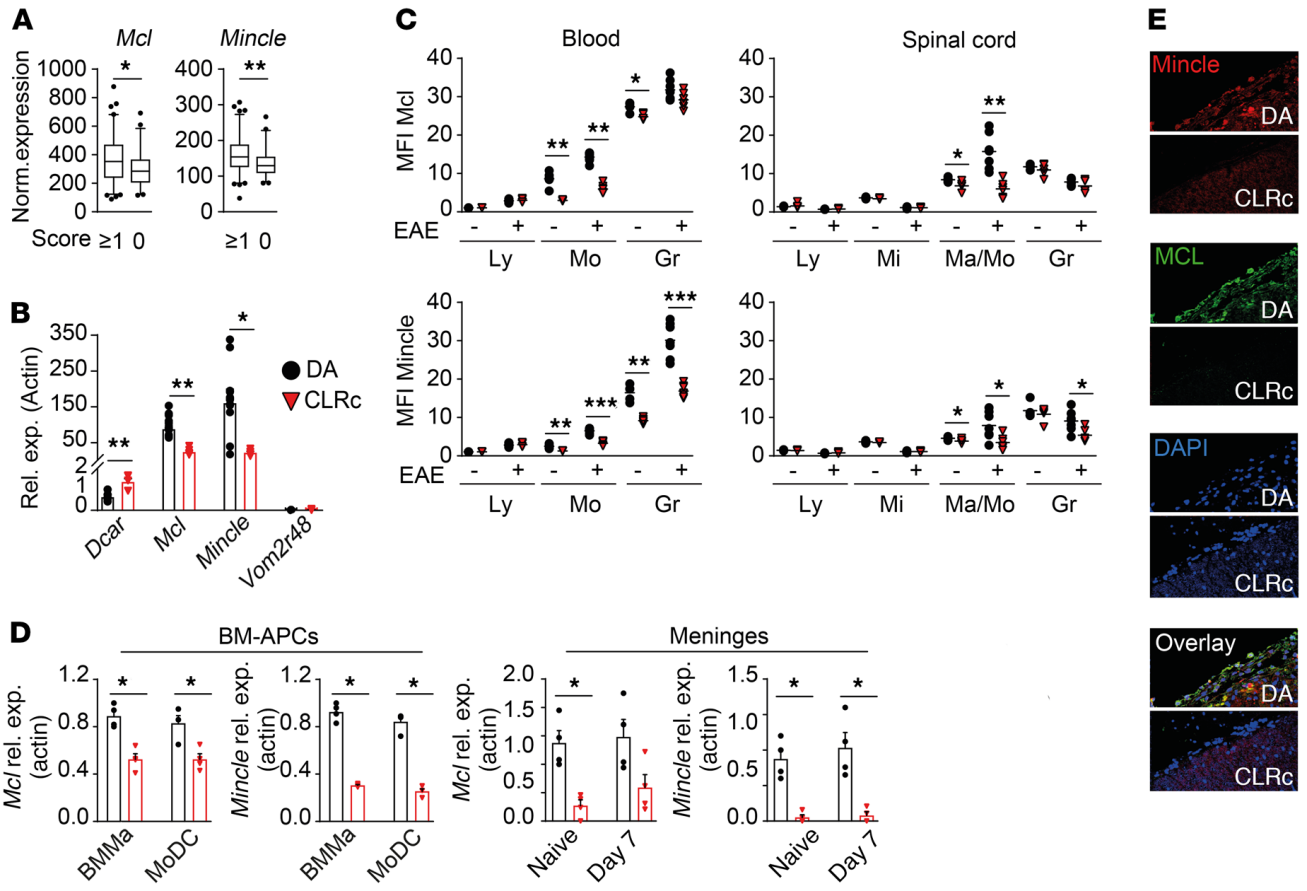
CLRc regulates expression of Mcl and Mincle in monocytes and macrophages. (**A**) Expression of *Mcl* and *Mincle* assessed by microarray in spleens of (DA × PVG) × DA backcrossed rats subjected to EAE, affected (*n* = 95, score ≥1) and nonaffected (*n* = 51, score 0). (**B**) Gene expression in DA (*n* = 6) and CLRc (*n* = 6) spleens determined by qPCR (representative of 2 experiments). (**C**) Flow cytometry analysis of cells isolated from blood and spinal cord of naive DA (*n* = 5) and CLRc (*n* = 5), as well as DA (*n* = 7) and CLRc (*n* = 6) rats on day 13 p.i. assessing Mcl and Mincle protein expression by mean fluorescence intensity (MFI) (representative of 2 experiments). Ly, lymphocyte; Mi, microglia; Mo/Ma, monocyte/macrophage; Gr, granulocyte. (**D**) qPCR analysis of *Mcl* and *Mincle* expression in BMMas and MoDCs (BM-APCs) derived in vitro and meninges from naive and 7-day-p.i. DA (*n* = 4) and CLRc (*n* = 4) rats (representative of 2 experiments). (**E**) Immunofluorescent staining of rat spinal cord 11 days p.i. Representative images of staining for Mcl, Mincle, and nuclei (DAPI) (original magnification, ×40). Data are presented as the mean ± SEM or box plots with whiskers representing 5th to 95th percentile. All comparisons were analyzed with the Mann-Whitney *U* test. *P < 0.05; **P < 0.01; ***P < 0.001.

Using sequencing and single-nucleotide polymorphism (SNP) typing, we determined that the CLRc region contains 4 genes of the CLR Dectin-2 family, i.e., *Dcar1*, *Mcl*, *Mincle*, and the pseudogene *Dectin-2p*, as well as the vomeronasal gene *Vom2r48* (Supplemental Table 1). We first examined the association of genes of the CLRc locus with different EAE disease parameters using gene expression data from splenocytes of (DA × PVG) × DA backcrossed rats (*n* = 150) subjected to EAE (27). Among the 5 investigated genes, significant associations with disease phenotype were only observed for *Mcl* and *Mincle* (Supplemental Table 2). The expression of both genes positively correlated with disease incidence (Figure 3A), maximal score, cumulative score, disease duration, terminal score, and maximal weight loss (Supplemental Table 2 and Supplemental Figure 3). We next assessed the expression of the CLRc genes in splenocytes of naive DA and CLRc rats by quantitative real-time PCR (qPCR) and determined a significantly reduced expression of *Mcl* (70%) and *Mincle* (85%) and increased expression of *Dcar1* (212%) in CLRc animals compared with DA rats (Figure 3B). Expression of *Vom2r48* was below the detection threshold and we failed to amplify *Dectin-2p* despite employing multiple strategies, which is not unusual for a pseudogene. Because the expression of *Dcar1* did not correlate with any phenotypic features of disease, the reduced expression of both *Mcl* and *Mincle* in immune cells is more likely to confer the protection to EAE observed in CLRc rats.

To further characterize the cellular compartment mediating the EAE phenotype, we examined expression of Mcl and Mincle on different immune cells in DA and CLRc rats. In the blood of naive animals, significantly fewer circulating Mcl*^+^* and Mincle*^+^* monocytes were observed together with reduced surface receptor expression on both monocytes and granulocytes in CLRc compared with DA rats (Figure 3C and Supplemental Figure 4). In the spinal cord, naive CLRc rats displayed a reduction in both frequency of Mcl*^+^* and Mincle*^+^* cells and relative receptor expression levels on infiltrating monocytes/macrophages. Similar results were observed in both the blood and spinal cord of EAE-affected animals, although only Mincle and not Mcl expression was affected in granulocytes during disease (Figure 3C and Supplemental Figure 4). No difference in the microglial compartment was observed in EAE animals, supporting the hypothesis that expression of the receptors on microglia is not likely to affect EAE disease outcome (Figure 3C and Supplemental Figure 4).

To analyze the effect of CLRc on tissue-resident and inflammatory myeloid cells we generated BM-derived macrophages (BMMas) and dendritic cells (MoDCs) (28), and observed reduced expression of *Mcl* and *Mincle* in CLRc cells compared with cells derived from DA rats (Figure 3D). Furthermore, *Mcl* and *Mincle* expression in the meninges ex vivo was significantly lower in naive and immunized CLRc rats (Figure 3D). This was reflected at the protein level, where 11 days p.i. receptor expression was only detected in DA rats but not in CLRc spinal cord meninges (Figure 3E), in concordance with Mcl^+^ and GFP^+^ cells only being detected in the meninges of DA-GFP → DA rats (Supplemental Figure 1J).

Taken together, these data indicate that the EAE protection conferred by the CLRc locus is associated with reduced expression of Mcl and Mincle*,* particularly in myeloid cells residing in the meninges.

### Impaired response to Mcl/Mincle ligands in CLRc myeloid cells affects CD4^+^ T helper cell recruitment and skewing.

**Figure 4:**
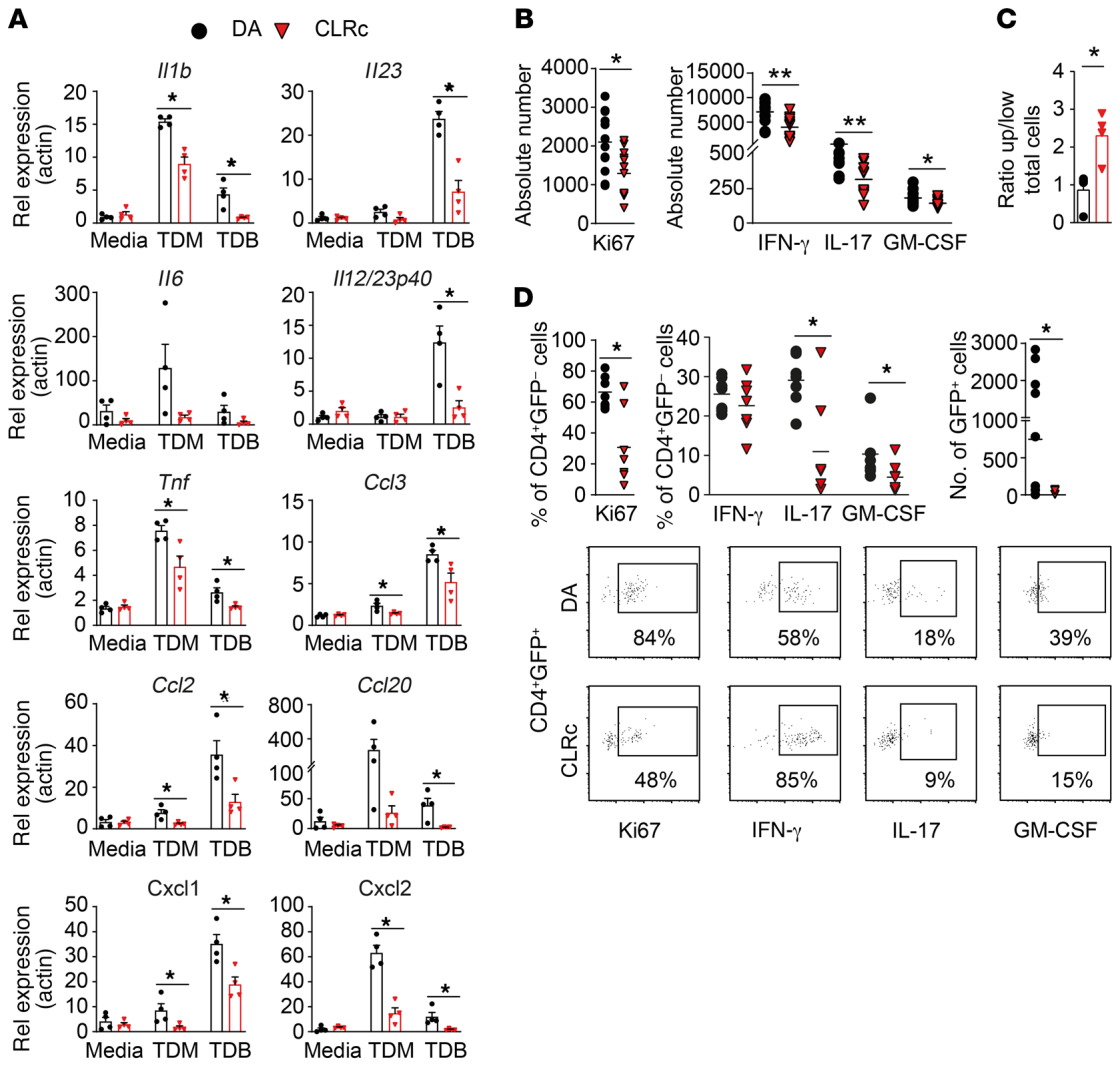
Attenuated response to Mcl/Mincle stimulation in CLRc BMMas resulting in altered CD4^+^ T cell extravasation and activation. (**A**) Bone marrow–derived macrophages (BMMas) from DA (*n* = 4) and CLRc (*n* = 4) rats were stimulated with receptor-specific ligands (TDM and TDB) for 18 hours (representative of 3 experiments). Genes downstream of the Mcl/Mincle pathway were analyzed by qPCR. (**B**) MOG-specific CD4^+^ effector cells were reactivated for 4 days with DA (*n* = 6) or CLRc (*n* = 6) BMMas at a ratio of 40:1 (T cell/BMMa) in the presence of MOG peptide. Flow cytometry analysis of CD4^+^ T cell assessing cytokine production and proliferation (representative of 2 experiments). (**C**) Transendothelial extravasation of MOG-specific CD4^+^ effector cells toward DA (*n* = 4) or CLRc (*n* = 4) BMMas. Flow cytometry analysis of CD4^+^ T cell assessing transmigration (representative of 2 experiments). (**D**) Adoptive transfer of GFP^+^ MOG-specific CD4^+^ effector cells injected i.v. into DA (*n* = 7) or CLRc (*n* = 7) recipients 6 days p.i. (representative of 2 experiments). Characterization of proliferation and cytokine production in both GFP^–^ as well as GFP^+^ infiltrating cells isolated from spinal cord on day 13 p.i. stimulated in vitro with PMA/ionomycin/brefeldin A for 5 hours. Data are presented as the mean ± SEM. All comparisons were analyzed with the Mann-Whitney *U* test. *P < 0.05; **P < 0.01.

To further investigate the functional consequences of low Mcl/Mincle levels, we assessed expression of genes known to be induced by the Mcl/Mincle signaling pathway in BMMas, MoDCs, and FLT3-derived BM dendritic cells (BMDCs) stimulated with Mcl/Mincle–specific ligands, TDM and TDB. All CLRc myeloid cell types showed a reduced expression of *Mcl*/*Mincle* (Supplemental Figure 5, A–C) and an impaired response to Mcl/Mincle ligand activation (Figure 4A and Supplemental Figure 5, D and E). More specifically, CLRc BMMas exhibited significantly lower expression of cytokines such as *Il1b*, *Il6*, *Il23*, *Il12p40*, and *Tnf* as well as the chemokines *Ccl2*, *Ccl3*, *Ccl20*, *Cxcl1*, and *Cxcl2* upon stimulation with TDM and/or TDB compared with DA cells. Similar results were obtained when stimulating MoDCs and BMDCs (Supplemental Figure 5, D and E). We also investigated the signals that regulated *Mcl*/*Mincle* expression and found TNF, and to a minor extent IL-1α and IL-1β, to upregulate both receptors (Supplemental Figure 5G).

T helper cell differentiation requires TCR activation (signal 1), costimulation (signal 2), and a third signal mediated by cytokines. We therefore performed in vitro T cell differentiation assays using the supernatants from TDM- or LPS-stimulated MoDCs as a surrogate for the third signal of T cell activation. While we did not detect any differences in cytokine production or proliferation of CD4^+^ T cells exposed to DA or CLRc supernatants from LPS-stimulated cells, supernatants from TDM-stimulated CLRc cells led to a reduced induction of IFN-γ and IL-17 together with a strong decrease in cell proliferation accompanied by increased IL-10 production (Supplemental Figure 5F). This reduced inflammatory profile did not stem from either TGF-β or IL-10 production by TDM-stimulated CLRc cells, since no difference in TGF-β and only very low production of IL-10 could be detected in the MoDC supernatants (Supplemental Figure 5H).

We thereafter explored whether the compromised response to Mcl/Mincle stimulation in myeloid cells could affect T cell recruitment, activation, and fate. We generated GFP^+^ MOG-specific resting effector T (GFP^+^ MOG-Teff) cells. GFP^+^ MOG-Teff cells were reactivated in vitro with either DA or CLRc BMMas to mimic the reactivation of pathogenic T cells in the CNS (Figure 4B). Coculture of GFP^+^ MOG-Teff with CLRc BMMas led to a reduced proliferation of T cells as well as reduced number of IFN-γ–, IL-17–, and GM-CSF–producing T cells compared with cells cultured with DA BMMas. We then assessed if CLRc BMMas could promote differential recruitment of GFP^+^ MOG-Teff cells across a CNS vascular endothelial cell layer, mimicking the meningeal blood–cerebrospinal fluid (blood-CSF) barrier, using a transwell migration assay. We found higher retention of GFP^+^ MOG-Teff in the upper chamber (higher ratio) when T cells were exposed to CLRc BMMas, whereas DA BMMas induced more extravasation through the endothelial layer (lower ratio) (Figure 4C). Finally, we transferred the GFP^+^ MOG-Teff cells into immunized DA or CLRc rats on day 6 p.i. and assessed T cell frequencies and activation at disease onset (day 13 p.i.). Similarly to EAE induction in unmanipulated DA and CLRc rats (Figure 2), we observed less proliferation as well as fewer IL-17– and GM-CSF–producing GFP^–^ T cells in the spinal cord of CLRc rats compared with DA (Figure 4D). Interestingly, we also recovered fewer GFP^+^ MOG-Teff cells in the CNS of CLRc rats and those cells proliferated less and also produced less IL-17 and GM-CSF (Figure 4D).

Altogether, our in vitro and in vivo findings indicate that reduced Mcl/Mincle expression results in impaired myeloid cell activation that leads to reduced T cell recruitment to the CNS and compromised T helper cell reactivation.

### Downregulation of Mcl and Mincle, and their endogenous ligand SAP130, contributes to EAE pathogenesis.

**Figure 5:**
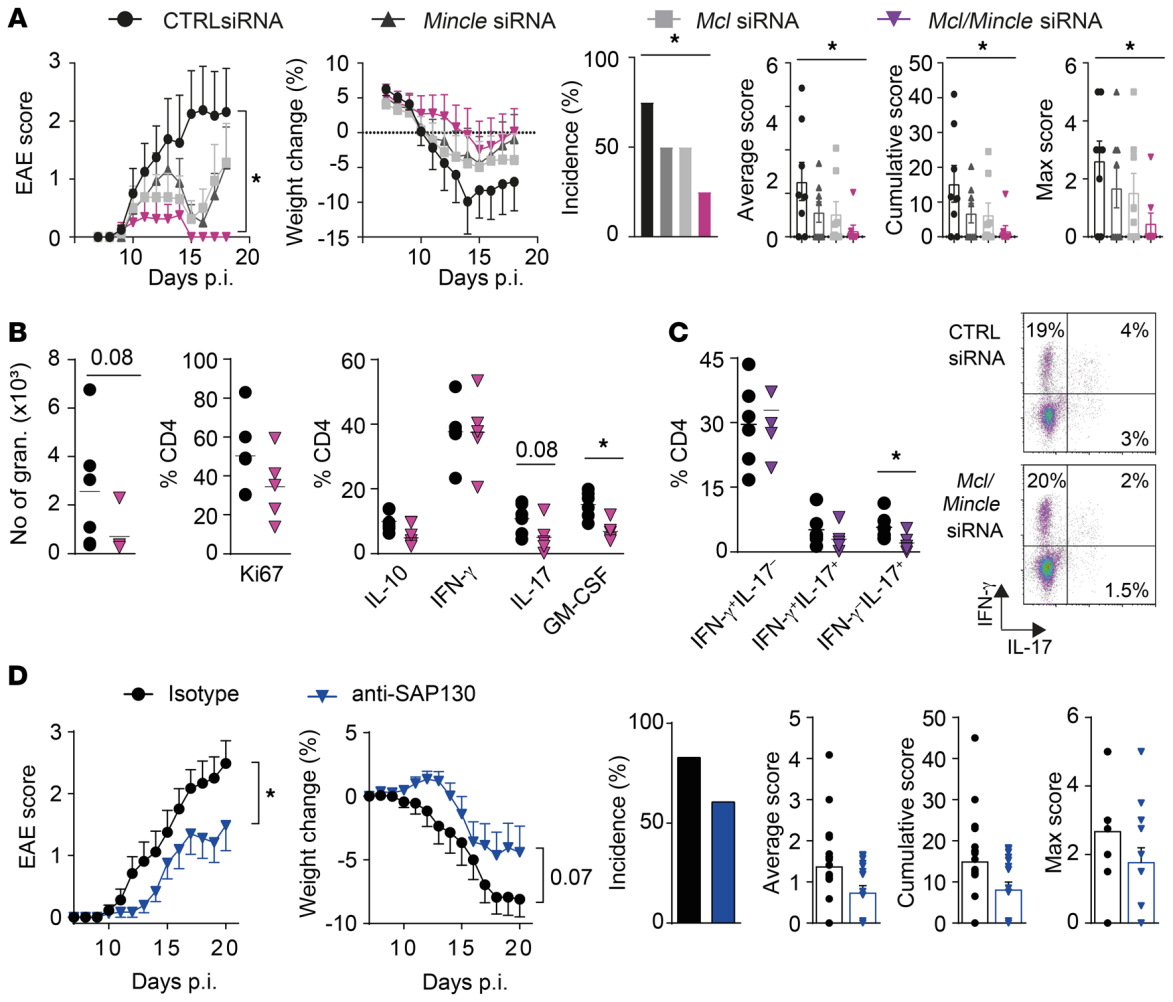
CNS-specific *Mcl*/*Mincle* silencing, as well as blockage of the endogenous ligand SAP130, protects from EAE. (**A**) MOG-immunized rats injected with *Mcl* (*n* = 8)*, Mincle* (*n* = 8)*, Mcl*/*Mincle* (*n* = 8), or scrambled control siRNA (*n* = 8) i.t. and i.c. at 7, 9, and 12 days p.i. were followed for clinical signs of EAE (representative of 3 experiments). (**B**) Characterization of proliferation and cytokine production of infiltrating cells isolated from spinal cord on day 12 p.i., stimulated in vitro with PMA/ionomycin/brefeldin A for 5 hours (proliferation and cytokine production) in rats injected with *Mcl*/*Mincle* (*n* = 5) or scrambled control siRNA (*n* = 6) and (**C**) quantification of IFN-γ– and IL-17–producing CD4^+^ T cells (representative of 2 experiments). (**D**) Clinical signs of EAE and disease parameters in DA rats treated with anti-SAP130 (*n* = 18) or rabbit IgG isotype control (*n* = 18) antibody i.t. and i.c. on days 2 and 7 p.i. (2 pooled experiments). Data are presented as the mean ± SEM. The following statistical tests were used: 1-way ANOVA with Dunnett’s multiple-comparisons test (**A**, for area under the curve [AUC] of clinical EAE and weight change), Mann-Whitney *U* test (**A**–**D** [for average, cumulative, and max EAE score], **B**, and **C**), unpaired 2-tailed *t* test (**D**, for AUC of clinical EAE and weight change), and χ^2^ test (**A** and **D**, for EAE incidence). *P < 0.05.

To confirm the impact of Mcl and Mincle expression on neuroinflammation in EAE, we performed in vivo CNS-specific knockdown of *Mcl* and *Mincle* expression. Infiltrating and resident cells in both spinal cord and the brain subarachnoid space were targeted via delivery of *Mcl*/*Mincle*–specific siRNA using intrathecal (i.t.) and intracisternal (i.c.) injections on days 7, 9, and 12 p.i. in disease-susceptible DA rats. The silencing efficiency of combined siRNA targeting of *Mcl* and *Mincle* was verified in vitro in BMMas, with a 50%–70% decrease in Mcl and Mincle surface expression compared with control siRNA (Supplemental Figure 6). Interestingly, rats treated with *Mcl*/*Mincle*–specific siRNA had a reduced EAE disease incidence resulting in lower cumulative and average scores as well as weight decrease compared with animals treated with control siRNA (Figure 5A). This was accompanied by a trend of decreased infiltration of granulocytes and reduced frequency of IL-17– and GM-CSF–producing CD4^+^ T cells in the CNS prior to disease onset (Figure 5, B and C). In rats treated with either *Mcl-* or *Mincle-*specific siRNA separately, we also observed a protection that was less robust compared with the effect of combined targeting of *Mcl* and *Mincle* (Figure 5A). Altogether, the results validate that altered expression of Mcl and Mincle modulates EAE susceptibility by affecting T cell reactivation in the CNS.

Mcl and Mincle can recognize both PAMPs and DAMPs. The danger molecule SAP130 was the first established endogenous ligand demonstrated to bind to Mincle (15) and elevated SAP130 levels have been implicated in several inflammatory experimental models (29, 30). We addressed the possible involvement of SAP130 in disease and hypothesized that blocking the protein in susceptible rats would affect EAE pathogenesis. Immunized DA rats were treated i.t. and i.c. with either anti-SAP130 or isotype control antibodies on day 2 and 7 p.i. Remarkably, blocking SAP130 in the target organ in vivo led to a reduction in EAE score as well as weight loss (Figure 5D). Additionally, a tendency for reduced incidence and average and cumulative score was observed.

Taken together, these results suggest that Mcl, Mincle, and their putative ligand SAP130 promote EAE pathogenesis.

### Increased activity of the MCL and MINCLE signaling pathway in patients with MS.

**Figure 6:**
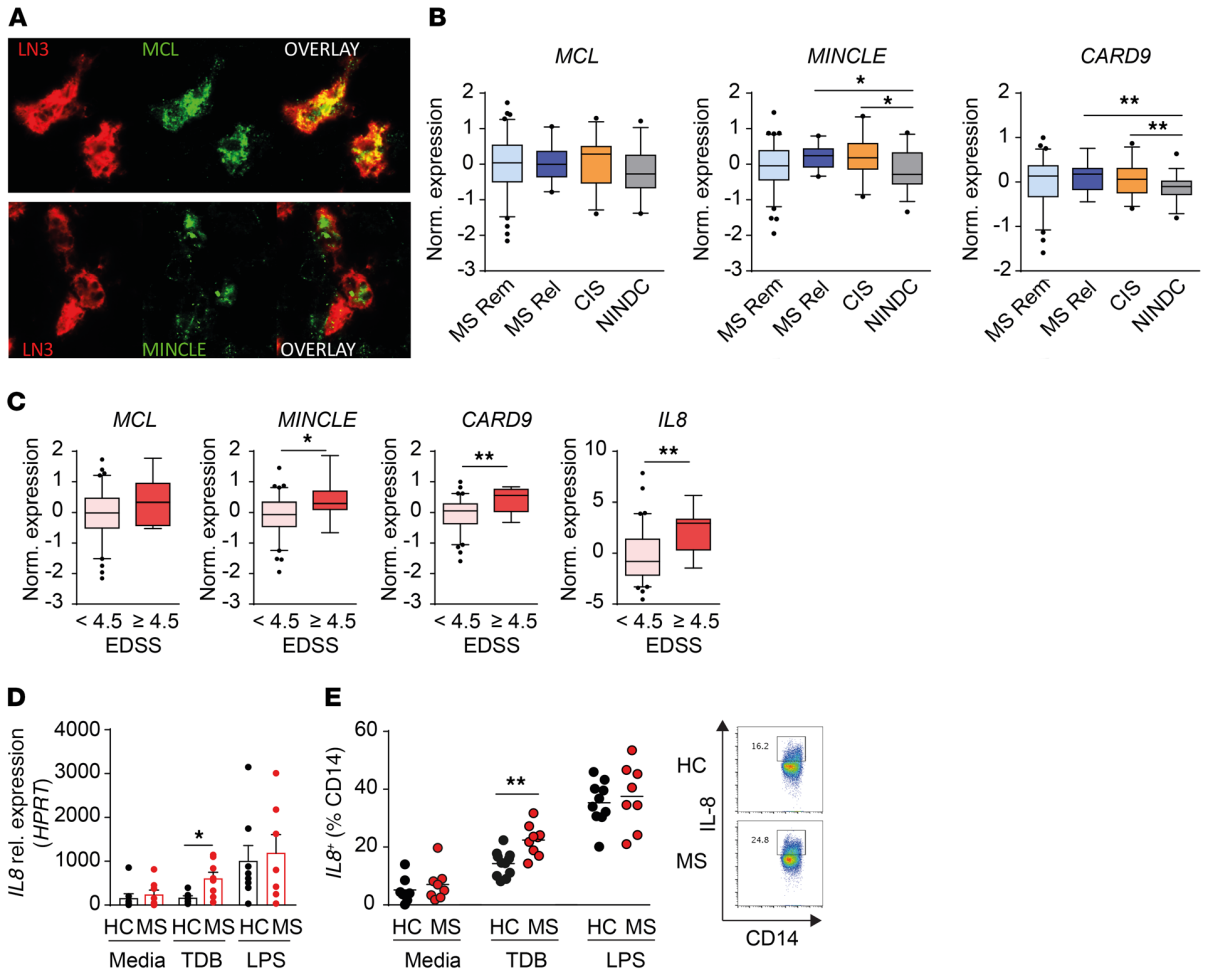
The MCL/ MINCLE signaling pathway is upregulated in MS patients and correlates with disease activity and progression. (**A**) Representative immunofluorescent staining of MS lesions: MCL or MINCLE (green), HLA-DR expression (LN3, red). Original magnification, ×40. (**B** and **C**) Gene expression analysis of PBMCs from MS patients and noninflammatory neurological disease controls (NINDCs) using RNA sequencing. (**B**) Expression of *MCL*, *MINCLE*, and *CARD9* comparing MS patients in remission (*n* = 73), MS patients in relapse (*n* = 14), CIS patients (*n* = 28), and NINDCs (*n* = 36). (**C**) Expression of *MCL*, *MINCLE*, *CARD9*, and *IL8* according to Expanded Disability Status Scale (EDSS) score. (**D**) PBMCs from RRMS patients in relapse (*n* = 9) and healthy controls (HC) (*n* = 11) were stimulated in vitro with TDB, LPS, or media. qPCR analysis of *IL8* expression of adherent CD14^+^ fraction after 24-hour stimulation. (**E**) Flow cytometry analysis of IL-8 production gated on CD14^+^ monocytes after 48-hour stimulation. Data are presented as the mean ± SEM or box plots with whiskers representing 5th to 95th percentile. The following statistical tests were used: 1-way ANOVA with Dunnett’s multiple-comparisons test (**B**), Mann-Whitney *U* test (**C** and **D**), and unpaired 2-tailed *t* test (**E**). *P < 0.05; **P < 0.01.

Our findings suggest a role for the C-type lectins Mcl and Mincle in the pathogenesis of EAE. To explore their potential relevance in human disease we first examined receptor expression in MS brain tissue both in active and chronic active lesions (Supplemental Table 3). Interestingly, both MCL and MINCLE expression was detected in active lesions and in the rim of chronic active lesions. Immunoreactivity of MCL and MINCLE localized on HLA-DR–immunopositive cells, indicating expression on APCs, primarily myeloid cells (Figure 6A and Supplemental Figure 7A). We thereafter determined the expression of genes involved in the MCL/MINCLE signaling pathway in circulating blood cells by analyzing the transcriptome of PBMCs in a large cohort of patients with MS, patients with clinically isolated syndrome (CIS), and noninflammatory neurological disease controls (NINDCs) (Supplemental Table 4). In accordance with previous studies reporting coregulation of *MCL* and *MINCLE*, we could detect a strong correlation between *MCL* and *MINCLE* expression in PBMCs from MS patients (Supplemental Figure 7B). Gene expression analysis revealed a significant increase in *MINCLE* and one of the downstream mediators, *CARD9*, in CIS patients and relapsing-remitting MS (RRMS) patients during relapse compared with NINDCs (Figure 6B and Supplemental Figure 7C).

We further investigated a putative association between the MCL/MINCLE pathway and MS disease severity by stratifying MS patients according to level of accumulated disabilities (Expanded Disability Status Scale, EDSS). We observed higher expression of *MINCLE*, *CARD9*, and *IL8* in patients that have higher disabilities (EDSS > 4.5) compared with less-affected patients (Figure 6C). Together, these results indicated a link between increased peripheral inflammatory activity and disease severity with the MCL/MINCLE signaling pathway in MS patients and prompted us to explore the impact of MCL/MINCLE activation in immune cells. To this end, we stimulated PBMCs isolated from RRMS patients during relapse and from healthy controls (Supplemental Table 5) with either TDB or LPS and examined the modulation of *MCL*/*MINCLE* expression as well as the downstream mediators *SYK*, *MALT1*, and *CARD9*, and effector cytokines IL-8, IL-6, and TNF (Figure 6, D and E, Supplemental Figure 7, D–G) (31). Interestingly, TDB stimulation resulted in a significant increase in IL-8 production at mRNA and protein levels, but not IL-6 or TNF, in monocytes from MS patients compared with healthy controls. Of note, there was no difference in *MINCLE* basal expression prior to stimulation between MS and healthy control monocytes, but there was an increased expression of *MCL*, *SYK*, and *MALT1* in MS patients (Supplemental Figure 7, D and E). Upon TDB stimulation, *MCL*, *MINCLE*, and *MALT1* were significantly upregulated in monocytes from MS patients (Supplemental Figure 7, D and E), with a similar trend observed for *SYK* and *CARD9*.

Altogether, our data strongly suggest an increased activity of the MCL/MINCLE signaling pathway in MS patients, which could play a role in the inflammatory response and disease progression.

## Discussion

Using a congenic rat strain with natural variation in expression of CLRs, we investigated the role of the PRRs Mcl and Mincle in EAE pathogenesis, and further examined the findings in human disease. Our data demonstrate that lower expression of Mcl and Mincle in the congenic strain protects them from development of MOG-EAE. Reduced receptor expression drastically affects the response of APCs to specific ligands and mitigates their ability to recruit and support a pathogenic Th17/GM-CSF phenotype. Moreover, we determined that MCL and MINCLE are expressed in MS lesions, that the signaling pathway is active in PBMCs of CIS and MS patients during relapse compared with controls, and that monocytes from MS patients responded more vigorously to specific receptor stimulation. Furthermore, expression of genes in the MCL/MINCLE pathway was increased in patients with higher disabilities, suggesting a role for this pathway in disease progression.

While PRRs, especially *MINCLE*, have been implicated in peripheral inflammatory disorders such as rheumatoid arthritis (32, 33), their role in CNS-related immune diseases such as MS remains elusive. Peripheral inflammation induces substantial changes in the CNS, particularly local neuroinflammation mediated by glial activation and subsequent production of proinflammatory factors such as TNF, IL-1β, and IL-6 cytokines (34, 35). Accordingly, studies have shown that following peripheral inflammation, recruitment of T cells and monocytes into the CNS and the resulting tissue damage is strongly influenced by local production of IL-6 and its effect on blood-brain barrier permeability (36, 37). Our data demonstrate that regulation of Mcl and Mincle in meningeal myeloid cells modulates T cell recruitment and reactivation in the CNS by a number of mechanisms that might include both proinflammatory cytokine and chemokine production, suggesting that these receptors would play a role in initiating or amplifying immune responses in the target organ. This is further supported by the increased expression of *MCL*, *MINCLE*, and downstream mediator *CARD9* observed in PBMCs of MS patients during active disease. Furthermore, we found that the MCL/MINCLE ligand TDB induces higher production of IL-8 by PBMCs from MS patients compared with controls, which is in accordance with previous data implicating the downstream mediators, CARD9 and SYK, in IL-8 production by human myeloid cells following TDB stimulation (31). Interestingly, animals with MOG_35–55_-specific CD4^+^ T cells (2D2 mice) display a higher incidence of spontaneous EAE disease following influenza respiratory infection when compared with noninfected mice, suggesting that the peripheral infection can trigger CNS immunosurveillance and disease (38). Consistent with this, epidemiological data have suggested that systemic infection either precedes or concurs with disease exacerbation in MS patients (39) and that stress may also be a contributing factor (40). Hence, stress or infections could induce expression of PRRs in CNS-innate cells of MS patients, promoting peripheral immune cell CNS patrolling, which would result in disease relapse. The protective effect of i.t and i.c. siRNA-mediated knockdown of *Mcl* and *Mincle* strongly supports a role for the receptors in the regions directly in contact with the CSF, such as the leptomeninges. Our findings demonstrating the expression of Mcl and Mincle in meningeal macrophages of EAE animals and receptors expression in MHC II^+^ cells in brain lesions of MS patients argues in favor of this hypothesis. Taken together, these data point to a possible role of CNS-intrinsic processes involving MCL, MINCLE, and their signaling pathways during the inflammatory phase of MS.

Our data provide in vitro and ex vivo evidence that Mcl and Mincle, expressed by monocyte-derived macrophages and/or DCs, shape the activation toward a Th17 and GM-CSF–producing T cell phenotype in EAE. While we also see an increased influx of granulocytes upon disease onset, the effect of these cells in further shaping the inflammatory response in the CNS is most likely secondary to T cell recruitment and activation, as it has been shown that neutrophils only acquire significant antigen-presenting capacity upon exposure to T cell–derived cytokines (41). Our findings agree with previous studies reporting an effect of Mincle on T helper cell differentiation, notably of Th17 cells (42), and a Mincle-associated Th17 response in vivo in the context of autoimmunity and sterile inflammation (43, 44). Notably, the mechanism of action for Mcl and Mincle appears highly important in light of the crucial role of Th17 cells and the cytokine GM-CSF in MS pathogenesis (21–26). Indeed, previous studies have determined an increased frequency of Th17 cells and GM-CSF–producing Th cells in the blood and CSF of MS and CIS patients during active disease (45, 46), and have further associated the frequency of effector memory Th17 cells as well as IL-17a levels with disease severity (EDSS) (45, 47). Similarly, we found that expression of genes from the MCL/MINCLE signaling pathway correlates with disease activity and severity.

The danger theory, proposed by Matzinger and colleagues (48), suggests that the immune system responds to intrinsic and extrinsic danger signals (PAMPs and DAMPs) by recognition through PRRs. DAMPs, sometimes referred to as alarmins, are endogenous molecules released upon cellular activation, stress, or damage and can induce activation of inflammatory pathways through PRRs in the absence of infection. Accordingly, several DAMPs have been implicated in the pathogenesis of autoimmune diseases such as rheumatoid arthritis, systemic lupus erythematosus, or systemic sclerosis (49, 50). Among the established endogenous ligands of Mincle, SAP130 has been reported to contribute to neuroinflammation in models of cerebral hemorrhage and traumatic brain injury (30, 51). Herein we showed that blocking SAP130 in the CNS ameliorated EAE, suggesting that SAP130 can act as an alarmin in neuroinflammation. However, whether SAP130 or other endogenous ligands play a role in MS pathogenesis remains to be explored.

In conclusion, our study reveals an important role of the myeloid CLR-mediated pathway in the amplification of autoimmune neuroinflammation upon initial CNS stress. In the future it will be crucial to define cellular and molecular drivers of the CLRc-mediated response using transgenic models as well as clinical samples in the context of genetic variants impacting the pathway. Of interest are also the mechanisms underlying CLR upregulation in MS patients and the characterization of the alarmins or other metabolic mediators that may impact the sterile inflammatory response. This is of particular importance, as most of the successful therapies for MS are able to prevent immune infiltration of the CNS but are limited in affecting subsequent steps of autoimmune attack in the target tissue.

## Methods

### Experimental design

The aim of this study was to investigate the potential role of PRRs in autoimmune neuroinflammation, more specifically the role of the CLRs Mcl and Mincle in MS, using both congenic rats expressing reduced levels of the receptors for in vivo and in vitro modeling as well as human samples from MS patients. For studies involving rats, littermate controls were used and in the event of treatment intervention, animals were randomly assigned into groups. In vivo and in vitro experiments were performed twice unless otherwise stated. For experiments involving human specimens, the number of samples included in each experiment was based largely on availability. Once experimental conditions were optimized, all data were included in the analysis. Data were thereafter only excluded upon technical experimental error. Animal experiments were performed blinded, as was the initial analysis of ex vivo and in vitro experiments.

### Animals

Inbred DA and PVG.1AV1 rats were originally obtained from the Zentralinstitut für Versuchstierzucht (Hannover, Germany) and Harlan UK Ltd, respectively. Separate lines have been established at Karolinska Institutet (DA/Kini and PVG.1AV1/Kini) (52). The CLRc strain stems from a natural recombination event between the DA strain and the APLEC strain, encompassing a larger congenic fragment from the PVG background (19, 53). Transgenic EGFP DA rats have been described previously (54, 55). Experiments were performed with both male and female rats ranging between 8 and 16 weeks of age, and experiments were always carried out with sex- and age-matched animals. All animals were bred and kept in 12-hour light/12-hour dark and temperature-regulated rooms. Rats were housed in polystyrene cages containing aspen wood shavings and had access to standard rodent chow and water ad libitum.

### Participants

Three cohorts of human samples were used in the study. Postmortem brain samples of both active lesions and normal-looking white matter were collected from MS patients in the Netherlands brain bank. PBMCs from CIS patients, untreated MS patients; RRMS patients in either relapse or remission, secondary progressive, primary progressive; as well as NINDCs were isolated for whole-genome RNA sequencing. For the human in vitro experiments, natalizumab-treated RRMS patients in remission and healthy controls were included.

### Genotyping

Genomic DNA was extracted using alkaline lysis of ear or tail biopsies. The TaqMan SNP assay was used to determine the genotype according to the manufacturer’s protocol (Thermo Fisher Scientific).

### EAE induction and experimental design

#### EAE induction.

EAE was induced as previously described (18). Rats were anesthetized with isoflurane (Forene) and injected subcutaneously in the dorsal tail base with 200 μL inoculum containing MOG (females 4–6 μg and males 10–15 μg) in PBS (Sigma-Aldrich) emulsified 1:1 with incomplete Freund’s adjuvant (Sigma-Aldrich). Rats were weighed and monitored for clinical signs of EAE daily from day 7 until termination of the experiment. Clinical scoring was performed according to a standard scale for EAE: 0, healthy; 1, tail weakness or tail paralysis; 2, hind leg paresis or hemiparesis; 3, hind leg paralysis or hemiparalysis; 4, tetraplegy; and 5, death. The following clinical parameters were assessed: incidence of EAE (clinical signs of EAE for at least 2 consecutive days), onset of EAE (the first day of clinical signs), maximum EAE score (the highest clinical score), cumulative EAE score (the sum of daily clinical scores), duration of EAE (number of days with EAE), weight change (calculated by subtracting the daily weight from the weight on day 7 and expressing the difference as a ratio of the weight on day 7), and maximum weight loss (calculated by subtracting the lowest weight during the experiment from the weight on day 7 and expressing the difference as a ratio of the weight on day 7).

#### BM chimeras.

Eight-week-old recipient animals (*n* = 7 or 8 per group) were lethally irradiated (twice with 550 Gy) and injected i.v. with freshly isolated BM cells (50 × 10^6^/300 μL) from donor animals (*n* = 3 per group). Rats were left to reconstitute for 8 weeks. Reconstitution efficiency was determined in blood by flow cytometry and animals were thereafter subjected to EAE.

#### Drug administration.

Immunized rats were randomly divided into 2 groups (*n* = 7 or 8 per group) and were treated with siRNAs, anti-SAP130, or respective controls by i.t. and i.c. (via cisterna magna) injections with 10 μL per injection. Rats were injected under 3.5%-isoflurane anesthesia, as previously described (56). The injections were performed with a 50-μL Hamilton syringe (Sigma-Aldrich). Animals were monitored daily and clinical signs of EAE were recorded as described above. For the siRNA treatment, groups received i.t. and i.c. injections of siRNAs against *Mcl*, *Mincle*, both *Mcl* and *Mincle*, or scramble siRNA, 5 μg/10 μL/injection in Accell siRNA delivery media (media and siRNA from Dharmacon) on day 7, 9, and 12 p.i. For the anti-SAP130 treatment, both groups received i.t. and i.c. injections of either anti-SAP130 or rabbit IgG (both from Abcam), 5 μg/10 μL/injection in PBS on day 2 and 7 p.i.

### Tissue collection

#### Lymph nodes and bones for BM extraction.

Rats were sacrificed with CO_2_ prior to dissection of inguinal lymph nodes and femoral bones and tissues were kept in PBS on ice until cell preparation.

#### CNS tissue.

Rats were anesthetized with isoflurane and perfused with PBS containing heparin at 2500 IU/L (Sigma-Aldrich) via the left ventricle. For flow cytometry, brains and spinal cords were excised and placed separately in 37% Percoll solution (Sigma-Aldrich) containing 50 U/mL DNase I (Roche Applied Science) on ice until further processing. Meninges from the spinal cord were separated and collected in cold PBS containing 2 mM EDTA (Sigma-Aldrich). For gene expression analysis, spinal cords or meninges were collected in RLT buffer containing dithiothreitol (DTT) (Sigma-Aldrich), dissociated using a tissue lyser (Qiagen), and supernatants were collected and frozen at –70°C until RNA preparation. For immunohistochemistry, rats were further perfused with 4% paraformaldehyde (PFA) (Histolab) and skulls and spines were dissected and kept in 4% PFA for 24 hours.

### Single-cell preparation and ex vivo culture

#### Lymph nodes.

Lymph nodes were mechanically dissociated and cells were spun and resuspended in complete media, RPMI supplemented with 5% FCS, 1% L-glutamine, 1% penicillin-streptomycin, 1% pyruvic acid (all from Sigma-Aldrich), and 50 mM 2-mercaptoethanol (Gibco-BRL). Subsequently, cells were plated either in 96-well V-bottom plates for further flow cytometry analysis or in 96-well U-bottom plates for recall stimulation (72 hours with 20 μg/mL MOG) and thereafter for flow cytometry analysis.

For in vitro T cell differentiation assay, lymph node single-cell suspensions were depleted of CD25^+^ cells (which include both recently activated T cells as well as Tregs) with anti-CD25-PE antibody followed by anti-PE beads and subsequently CD4^+^ cells were positively sorted using anti-CD4 beads (Miltenyi Biotec). Resting CD4^+^ T cells were added to 96-well flat plates precoated with anti-CD3 (1.25 mg/mL, BD Pharmingen), and cultured with supernatant from stimulated MoDCs and soluble CD28 (1 mg/mL, BD Pharmingen) for 4 days. Intracellular cytokine production was assessed by flow cytometry.

#### CNS cells.

Brain and spinal cord were dissociated with a glass homogenizer in 37% Percoll solution and underlaid with 70% Percoll solution. After centrifugation at 1000 *g* for 30 minutes, the intermediate layer was carefully collected and further diluted in HBSS (Sigma-Aldrich) prior to a 15-minute centrifugation at 600 *g*. Cell pellets were resuspended in PBS (Life Technologies) and further used for flow cytometry analysis. Meninges were incubated at 37°C for 20 minutes in PBS/EDTA and then mechanically dissociated through a 40-μm cell strainer. Cells were spun and resuspended in PBS until staining for flow cytometry.

#### BM-derived cells.

BM was flushed out of femurs with PBS. BM single-cell suspensions were used for injection in BM chimeras or plated to prepare BM APCs. BM APCs were prepared as described previously with modifications (28, 57). Briefly, BM cells were cultured in complete DMEM (Sigma-Aldrich) supplemented with 5% FCS, 1% L-glutamine, 1% penicillin-streptomycin, 1% pyruvic acid (all from Sigma-Aldrich), and 50 mM 2-mercaptoethanol (Gibco-BRL). This medium was further supplemented with a combination of 20 ng/mL rat recombinant (r) rGM-CSF and 5 ng/mL rat rIL-4 (to obtain MoDCs), with 200 ng/mL rhFLt3L (to obtain BMDCs), or with 20 ng/mL rat rM-CSF (to obtain BMMas) for 9 or 7 days respectively (all cytokines were purchased from PeproTech). BMMas and MoDCs (1 × 10^5^ cells/well) were stimulated in a 48-well plate with complete media (control well), 10 μg TDM (Innaxon), 10 μg TDB (InvivoGen), 100 ng/mL LPS, or with various concentrations of cytokines (TNF, IL-1β, IL-6, all from PeproTech). TDM or TDB was resuspended in isopropanol at 0.1 mg/mL and each well was coated with 100 μL and left to evaporate in a laminar-flow cabinet. Isopropanol was added to the control wells. Cell supernatants were used for T cell differentiation assay. Adherent cells were lysed in RLT containing DTT for gene expression analysis. For immunofluorescence (IF), DA-GFP BMMas were plated in culture slides and stimulated with cytokines for different time points. Cell were immediately fixed with 4% PFA and stained for IF.

#### In vitro siRNA-mediated MCL/Mincle silencing.

*Mcl* and *Mincle* expression was reduced using siRNA in vitro. *Mcl*- and *Mincle*-specific siRNAs or control scramble siRNA (Dharmacon) were prepared immediately prior to administration in Accell siRNA delivery media (Dharmacon), following the manufacturer’s instructions. BMMa cells (1 × 10^5^ cells/well in a 48-well plate) were treated with 6 μg of scrambled siRNA, pooled *Mcl*- and *Mincle*-specific siRNAs (3 μg each), or 6 μg scramble-GFP siRNA, and harvested 72 hours later in PBS/EDTA prior to flow cytometry.

#### Generation of the GFP^+^ MOG_91–108_-specific T cells.

DA-GFP rats were immunized with 100 μg MOG_91–108_ in 200 μg CFA. After 7 days, a single-cell suspension from draining lymph nodes was restimulated with 20 μg/mL MOG_91–108_ at a concentration of 1 × 10^7^ T cells/mL in RPMI supplemented with 5% FCS, 1% L-glutamine, 1% penicillin-streptomycin, 1% pyruvic acid (all from Sigma-Aldrich), and 50 mM 2-mercaptoethanol (Gibco-BRL) for 4 days at 37°C and 5% CO_2_. Dead cells were removed with Ficoll (Ficoll Paque Plus, GE healthcare) and live cells cultured for 5 days at a concentration of 2 × 10^5^ cells/mL in complete RPMI with IL-2 (20% filtered mixed leukocyte antigen culture supernatant).

#### GFP^+^ MOG_91–108_-specific T cells and BMMa coculture.

BMMa cells were plated at 6.25 × 10^3^ cells/well in 96-well U-bottomed plates with 20 μg/mL MOG_91–108_ and left to rest for 24 hours, after which 2.5 × 10^5^ MOG_91–108_-specific T cells were added to each well and incubated at 37°C and 5% CO_2_ for 4 days. All conditions were made in duplicate.

### Transwell experiments

#### Endothelial cell culture.

Endothelial cell culture followed a previously described protocol (58) with adaptations for rat brain endothelia. DA rat brains were extracted and kept in cold dissection media (HBSS supplemented with 7.5% BSA and 1% penicillin-streptomycin). Brains were processed to remove the cerebellum and optic nerves, bisected, forebrains were separated, and forebrain meninges were then removed under a stereomicroscope and the myelin duvet removed from the forebrain interior to obtain the cortex. The cortex was transferred to L15 media containing papain (Sigma-Aldrich) and DNAse I (Calbiochem), mechanically dissociated by trituration, and left to digest for 1 hour at 37°C. The enzymatic reaction was stopped with HBSS containing 10% FCS. The pellet obtained was resuspended in 1× HBSS containing 22% BSA and separated using density-dependent centrifugation at 800 *g* for 15 minutes. The resulting pellet containing the brain microvessels was washed and added to complete rat brain endothelial growth culture media (cRBEGM, Sigma-Aldrich) containing 10% FCS, 1% penicillin-streptomycin, 5 ng/mL hFGF, 20 ng/mL hVEGF, and 5 ng/mL rEGF and plated in a T75 flask precoated with 20 μg/mL type IV collagen and 20 μg/mL fibronectin (all from R&D Systems) and left to incubate at 37°C and 5% CO_2_. After 12 hours, and again after 8 hours, the medium was refreshed with cRBEGM, the second time containing 4 μg/mL puromycin and left for 5 days at 37°C and 5% CO_2_. The medium was refreshed with cRBEGM containing 0.5 μg/mL puromycin for another 7 days, after which it was replaced with cRBEGM with no puromycin. Growth was monitored to check for endothelial cell confluence.

#### Transwell migration assay.

Endothelial cells were detached and harvested using Accutase 1× solution in PBS at a ratio of 1:1. Pelleted cells were resuspended in cRBEGM at a concentration of 3 × 10^5^ cells/mL. Transwell plate bottoms were seeded with 3 × 10^4^ BMMa cells from either DA or CLRc resuspended in complete DMEM and allowed to settle for 3 hours at 37°C and 5% CO_2_. The transwell inserts were prepared by coating with 100 μL of fibronectin at 20 μg/mL for 3 hours at 37°C and 5% CO_2_ and then seeded with 100 μL of 3 × 10^5^ cells/mL rat brain endothelial cells, mounted on the wells containing the macrophages, and allowed to rest for 24 hours at 37°C and 5% CO_2_ to achieve confluence. After incubation, 3 × 10^6^ MOG_91–108_-specific T cells were added in complete RPMI to each insert and the wells were allowed to incubate for 16 hours at 37°C and 5% CO_2_, after which media from the upper well and the lower well were removed and cells stained for flow cytometry.

### Flow cytometry

The staining procedures were performed by following the manufacturers’ protocols. Briefly, single-cell suspensions were incubated in 2% mouse serum (to block Fc receptors), and then washed and incubated with antibodies for cell surface expression. Mcl-A488 and Mincle-A647 antibodies were in-house and have been validated previously (20). All other antibodies were obtained from BD Biosciences, eBioscience, or ABDserotec: CD25 (clone OX-39), CD134 (clone OX-40), CD44 (clone OX-49), MHC II (RT1B clone OX-6), CD45 (clone OX-1, catalog 561588, BD), CD11b (clone WT.5, catalog 562108, BD), CD4 (clone OX-35, catalog 565432, BD), and granulocyte marker (clone RP-1, catalog 550002, BD). Dead cells were excluded using yellow fluorescent LIVE/DEAD marker (Molecular Probes). All stainings, washing steps, and acquisition were performed in cell staining buffer (BioLegend). For intracellular staining, cells were stimulated for 5 hours with phorbol 12-myristate 13-acetate (PMA) (50 ng/mL, Sigma-Aldrich), ionomycin (1 μg/mL, Sigma-Aldrich) and brefeldin A (GolgiPlug) (1 μL/mL, BD Biosciences). Cells were blocked with 2% mouse serum and stained for cell surface markers with yellow fluorescent LIVE/DEAD marker prior to washing, fixation, and permeabilization. Cells were then stained for intracellular proteins with antibodies specific for GM-CSF (MP1-22E9, eBioscience, catalog 12-7331-82), IL-17a (eBio17B7, eBioscience, catalog 45-7177-82), IL-10 (A5-4 BD Biosciences, catalog 562156), IFN-γ (DB-1, BD Biosciences, catalog 562213), and Ki67 (B56, BD Biosciences, catalog 561281). Acquisition was performed using a Gallios flow cytometer and results were analyzed using Kaluza flow analysis software (both from Beckman Coulter).

### qPCR and microarray

#### qPCR.

Total RNA was extracted from cell lysates in RLT containing DTT using an RNeasy Mini kit (Qiagen) with the QiaCube according to the manufacturer instructions. cDNA was subsequently synthesized using an iScript kit (Bio-Rad). qPCR was performed on a Bio-Rad CFX384/C1000 Real-Time Detection System with a 3-step PCR protocol using SYBR Green fluorophore. Relative quantification of gene expression was calculated using the standard curve method with fold expression normalized relative to the endogenous control gene *ACTB* (β-actin) or *HPRT* (hypoxanthine-guanine phosphoribosyltransferase).

#### Microarray.

Expression of *Mcl* and *Mincle* assessed by microarray in spleens of (DA × PVG) × DA F1 rats subjected to EAE, as previously described (27). The following settings for analysis were used: Summarization: probe logarithmic intensity error (PLIER) described in the Guide to probe logarithmic intensity error (PLIER) estimation (http://www.affymetrix.com). Background correction: PM-GCBG. Normalization: global median. The microarray data are available in MIAME-compliant (minimal information about a microarray experiments) format at the ArrayExpress Database (http://www.ebi.ac.uk/arrayexpress) under accession code E-MTAB-784.

### Histopathological analyses and immunohistochemistry

Histopathological analysis was carried out using Luxol fast blue and hematoxylin and eosin staining. PFA-fixed 3- to 5-mm-thick paraffin-embedded sections of the brain and spinal cord were dewaxed in xylol, rehydrated, and stained with hematoxylin and eosin (H&E) and Luxol fast blue to assess inflammation and demyelination, respectively. The inflammatory index and demyelination score were determined by the number and size of demyelinated lesions of each rat on an average of 10 complete brain and spinal cord cross sections as previously described (59).

For immunohistochemical analyses of rat samples, whole spines from PFA-perfused animals were collected and kept in PFA overnight. After washing with PBS, samples were placed in 20N EDTA to decalcify the bones for 2 weeks and then placed in 30% sucrose solution for 1 week prior to freezing and sectioning onto superfrost glass slides. After rehydration in PBS, sections were stained with fluoromyelin (Invitrogen) according to the manufacturer’s protocol. They were then stained for microglia (anti–Iba-1, Wako Chemicals, catalog 019-19741), astrocytes (anti-GFAP, Dako, catalog M0761), or T cells (anti-CD4, Bio-Rad Serotec, catalog MCA55A647) followed by secondary antibody staining. For Mcl (anti-clec4d, Abcam, catalog ab175021) and Mincle (16E3, Novus Biological, catalog NBP1-49311AF488) staining, sections were subjected to antigen retrieval in target retrieval solution low pH (Dako) prior to staining. All washing steps were performed in TNT buffer (0.1 M Tris-HCl, pH 7.5, 0.15 M NaCl, and 0.05% [v/v] Tween 20).

### Human studies

#### Immunohistochemical analysis.

Brain samples (*n* = 5) containing active and chronic active lesions were obtained from 3 MS patients in collaboration with the VUmc MS Centrum Amsterdam and the Netherlands Brain Bank. Active lesions were characterized by abundant infiltrated macrophages and microglial cell activation throughout the lesion and overt demyelination. Chronic active lesions were fully demyelinated and contained a rim of macrophages and activated microglia. Detailed clinical data are summarized in Supplemental Table 3. Formalin-fixed paraffin-embedded tissue was stained as described previously (60). In short, after deparaffinization and antigen retrieval, sections were incubated overnight with anti-MINCLE or anti-MCL (1:100) and anti–HLA-DR (anti-LN3, 1:100) followed by incubation with Alexa Fluor 488/594–labeled secondary antibodies (1:200; Molecular Probes) and analyzed by confocal microscopy (Leica DMI 6000 SP8).

#### Expression analysis.

PBMCs were collected from 102 MS patients, 28 CIS patients, and 36 NINDCs, diagnosed with, e.g., neuralgia, paresthesia, sensory symptoms, vertigo, and tension headache, between 2001 and 2010 at the Neurology Clinic of the Karolinska University Hospital, Solna, Sweden (61). At the time of sample collection, 14 RRMS patients were in relapse and 73 were in remission, and the remaining patients were diagnosed with secondary progressive MS (*n* = 8) or primary progressive MS (*n* = 7). mRNA was extracted from samples and cDNA libraries were prepared using an Illumina TruSeq kit and sequenced on an Illumina HiSeq 2000 instrument, as previously described (61).

For the RNA sequencing, we obtained paired-end reads with a length of 100 bp with an average sequence depth of 36 million reads per sample. The reads were mapped to the *H*. *sapiens* reference genome (NCBI v37, hg19) using STAR aligner and the HTSeq tool was used to quantify counts per gene, applying the default parameters in each case. The conditional quantile normalization (CQN) method was used to normalize the count data sets and to account for the GC-content bias. Implementing a component-based analysis, we regressed out the effects of batch of RNA sequencing library preparation from the normalized (CQN) count data. Residual values obtained after batch-effect correction was used for differential expression. The RNA sequencing data will be made available from the corresponding author upon request and signature of data transfer agreement.

#### Ex vivo PBMC culture.

RRMS patients treated with natalizumab (*n* = 9) and healthy donors (*n* = 11) were recruited and PBMCs were isolated by density gradient centrifugation using Ficoll-Paque plus (GE Healthcare) and stored in liquid nitrogen until use. Upon thawing, cells were washed, counted, and resuspended in RPMI medium supplemented with 20% FCS, L-glutamine, and penicillin-streptomycin. Cells were plated at 2 × 10^6^ cells/mL and stimulated with 40 μg plate-bound TDB, 50 ng soluble LPS, or wells were treated with isopropanol prior to air drying as negative controls. Cells were cultured for 48 hours and treated with GolgiPlug (BD Biosciences) 5 hours prior to termination of the experiment. Cells were stained for MCL (clone 9B9, catalog 360204) and isotype (clone MPC-11, catalog 400314) expression prior to in vitro stimulations (BioLegend). Upon stimulation, cells were stained with LIVE/DEAD stain (Thermo Fisher Scientific, catalog L34976), CD14 (clone HCD14, catalog 325608), IL-8 (clone E8N1, catalog 511406), IL-6 (clone MQ2-13A5, catalog 501114), TNF (clone Mab11, catalog 502926), or isotype for cytokines (clone MOPC-21, catalog 400108) (BioLegend).

### Statistics

The 1-way ANOVA with Dunnett’s multiple-comparisons test was used to compare area under the curve (AUC) of clinical EAE course and weight change as well as RNA sequencing data, when more than 2 groups were compared. The Kruskal-Wallis test with Dunn’s multiple-comparisons test was used to compare all EAE score–based variables (average, cumulative, and max EAE score) and histopathological variables, when more than 2 groups were compared. The unpaired 2-tailed *t* test was used to compare the AUC of clinical EAE course and weight change when 2 groups were compared. The Mann–Whitney *U* test was used to compare all EAE score–based variables (average, cumulative, and max EAE score) as well as microarray, flow cytometry, qPCR, and ELISA data, when 2 groups were compared. The χ^2^ test was used when analyzing the differences in EAE incidence. Values are expressed as the mean ± SEM or box plots with whiskers representing the 5th to 95th percentile. Prism 5.0 software (GraphPad) was used for statistical analyses and *P* values less than 0.05 were considered significant.

### Study approval

All animal experiments in this study were performed in accordance with the guidelines from the Swedish National Board for Laboratory Animals and European Community Council Directive (86/609/EEC), and approved by the North Stockholm Animal Ethics Committee (Stockholms norra djurförsöksetiska nämnd, Stockholm).

The human studies were approved by the Regional Ethics Committee (Stockholm, Sweden). All subjects in the RNA sequencing and in vitro monocyte stimulations provided written informed consent. For the brain cohort, all donors, or their next of kin, had given informed consent for brain autopsy and use of their brain material and clinical information for research purposes.

## Author contributions

The studies were designed by MN with contributions from SB, AOGC, and MJ. MN, SB, SF, LK, AW, MZA, EP, JHM, WE, FM, HYC, VM, JVH, and AOGC conducted experiments and acquired data. MN, SB, MZA, TJ, ML, JVH, MJ, and IK analyzed the data. HMR, MRD, JVH, and TO provided reagents for the experiments. MN wrote the manuscript with contributions from SB, LK, RAH, TO, AOGC, and MJ.

## Supplementary Material

Supplemental data

## Version 1 11/14/2019

In-Press Preview

## Version 2 01/13/2020

Electronic publication

## Version 3 02/03/2020

Print issue publication
