# Supplementary material for: C-type lectin receptors Mcl and Mincle control development of multiple sclerosis–like neuroinflammation
Source: J Clin Invest. 2020 Jan 13;130(2):838–52. doi: 10.1172/JCI125857 (PMC6994148; doi:10.1172/JCI125857)
Supplement: Supplemental data [file jci-130-125857-s201.pdf]

Supplementary Materials:

Figure S1. The protective effect of CLRc is dependent on infiltrating immune cells, but not on resident microglia.

Figure S2. Gating strategies for rat and human flow cytometry.

Figure S3. Correlation between *Mcl/Mincle* expression and clinical EAE parameters.

Figure S4. Reduced frequency of Mcl- and Mincle-expressing cells in CLRc congenic rats.

Figure S5. CLRc bone-marrow-derived macrophages and dendritic cells have a reduced response to Mcl/Mincle stimulation.

Figure S6. Evaluation of siRNA silencing of *Mcl* and *Mincle* in vitro.

Fig. S7. Expression of the MCL/MINCLE signaling pathway in MS patients.

Table S1. Genotyping of the CLRc region on rat chromosome 4.

Table S2. Correlation between CLR gene expression in spleen and different EAE phenotypes 35 days after EAE induction in (DAxPVG)xDA backcross rats.

Table S3. Baseline characteristics of the human brain cohort.

Table S4. Baseline characteristics of the human RNA-sequencing cohort.

Table S5. Baseline characteristics of the human in vitro stimulation cohort.

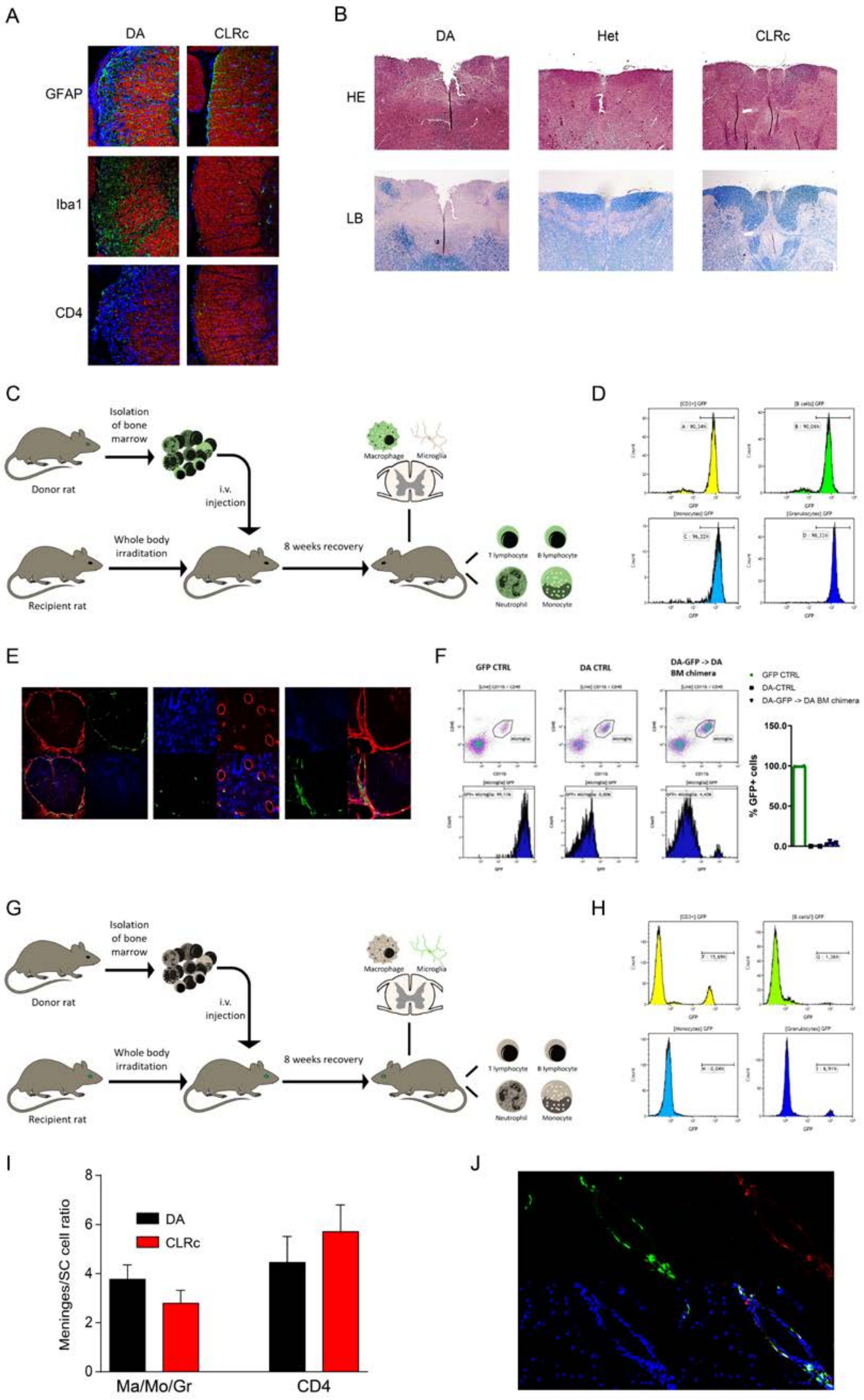

Fig. S1. The protective effect of CLRc is dependent on infiltrating immune cells but not microglia. (**A**) Immunofluorescent staining of DA and CLRc spinal cord day 24 p.i. Representative images of astrocytes (GFAP, green), microglia/macrophages (Iba1, green), and

T cells (CD4, green) along with myelin (fluoromyelin, red) and nuclei (DAPI, blue) (magnification 20X). (**B**) Histopathological analysis of brain (medulla) at day 29 p.i. Representative images of H&E and Luxol fast blue (LB) stainings (magnification 40X). (**C**) Schematic illustration of the bone marrow (BM) transfer (DA-GFP BM -> DA or CLRc) (**D**) Representative flow cytometry analysis of blood cells from BM transferred rats assessing level of reconstitution in DA-GFP -> DA rats at 8 weeks post transfer. (**E**) Immunofluorescent staining of sections from naïve BM-transferred rats assessing the replacement of meningeal and perivascular macrophages with donor BM-derived cells. BM-derived cells (GFP, green), meninges and blood vessels visualized by basal lamina staining (laminin, red), cell nuclei (DAPI, blue). Magnification for each panel, from left to right: 10X, 40X and 40X. (**F**) Flow cytometry analysis of spinal cord microglia from BM transferred rats assessing level of reconstitution in DA-GFP -> DA rats at 8 weeks post transfer. *Left*, representative flow cytometry plots. *Right*, quantification of GFP^+ microglia for DA-GFP (*n*=2), DA (*n*=2) and DA-GFP -> DA rats (*n*=4). (**G**) Schematic illustration of BM-transfer DA or CLRc BM -> DA-GFP. (**H**) Representative flow cytometry analysis assessing reconstitution efficiency in DA -> DA-GFP. (**I**) Ratio of the numbers of monocytes/macrophages/granulocytes and $CD4^+$ T cells infiltrating the meninges compared to spinal cord prior to EAE onset. Data are presented as mean ± SEM. (**J**) Immunofluorescent staining of immunized asymptomatic DA-GFP -> DA spinal cord day 31 p.i. assessing expression of Mcl (red) in GFP cells present in the meninges, nuclei stained with DAPI (blue) (magnification 40X).

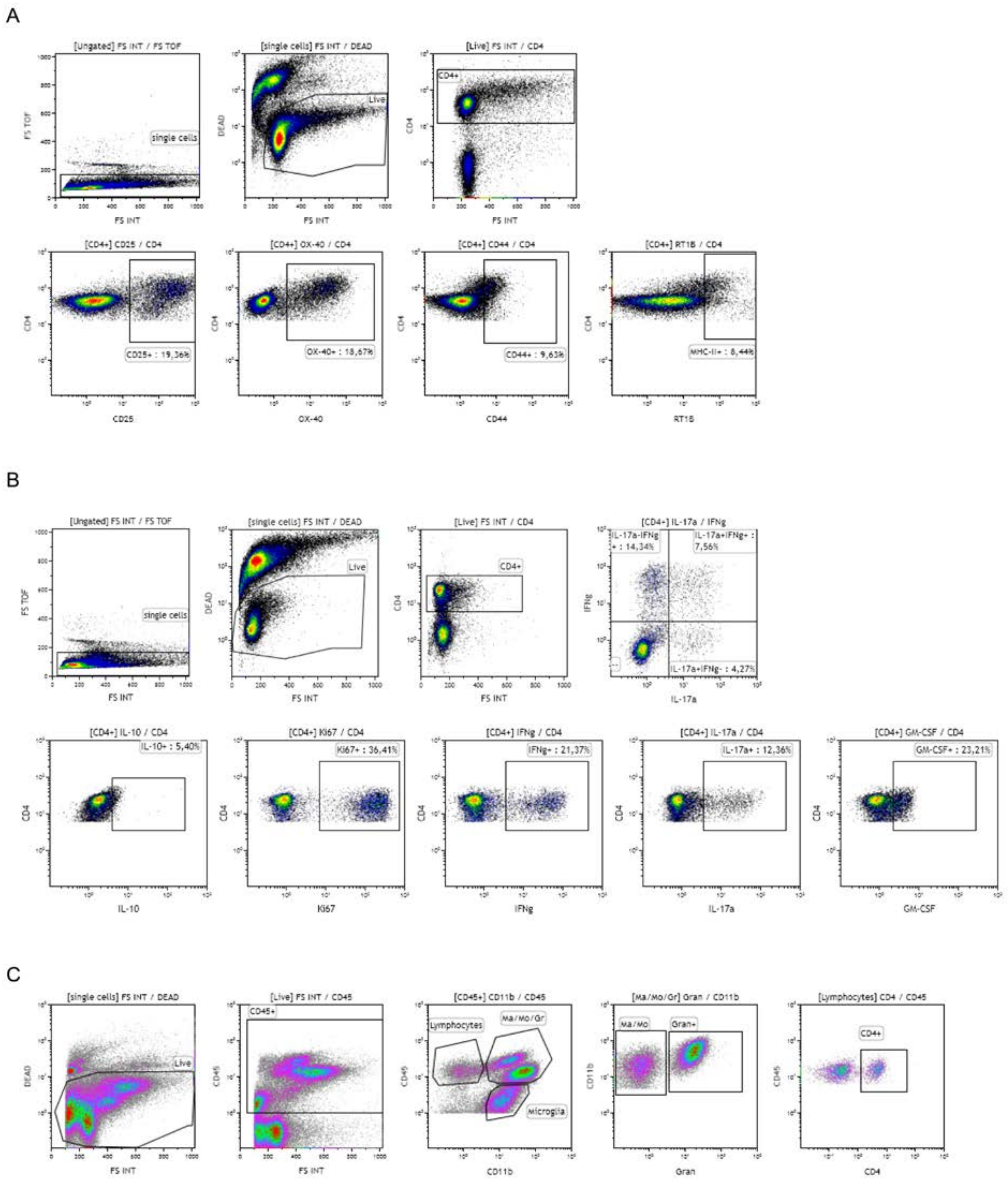
A
[Ungated] FS INT / FS TOF
single cells
[single cells] FS INT / DEAD
Live
[Live] FS INT / CD4
CD4+
FS INT
FS TOF
DEAD
CD4
[CD4+] CD25 / CD4
CD25+ : 19,36%
[CD4+] OX-40 / CD4
OX-40+ : 18,67%
[CD4+] CD44 / CD4
CD44+ : 9,63%
[CD4+] RT1B / CD4
MHC-II+ : 8,44%
CD25
OX-40
CD44
RT1B
B
[Ungated] FS INT / FS TOF
single cells
[single cells] FS INT / DEAD
Live
[Live] FS INT / CD4
CD4+
[CD4+] IL-17a / IFNg
IL-17a-IFNg+ : 14,34%
IL-17a+IFNg+ : 7,56%
IL-17a+IFNg- : 4,27%
IFNg
IL-17a
[CD4+] IL-10 / CD4
IL-10+ : 5,40%
[CD4+] Ki67 / CD4
Ki67+ : 36,41%
[CD4+] IFNg / CD4
IFNg+ : 21,37%
[CD4+] IL-17a / CD4
IL-17a+ : 12,36%
[CD4+] GM-CSF / CD4
GM-CSF+ : 23,21%
IL-10
Ki67
IFNg
IL-17a
GM-CSF
C
[single cells] FS INT / DEAD
Live
[Live] FS INT / CD45
CD45+
[CD45+] CD11b / CD45
Lymphocytes
Ma/Mo/Gr
Microglia
[Ma/Mo/Gr] Gran / CD11b
Ma/Mo
Gran+
[Lymphocytes] CD4 / CD45
CD4+
FS INT
CD11b
Gran
CD4
CD45

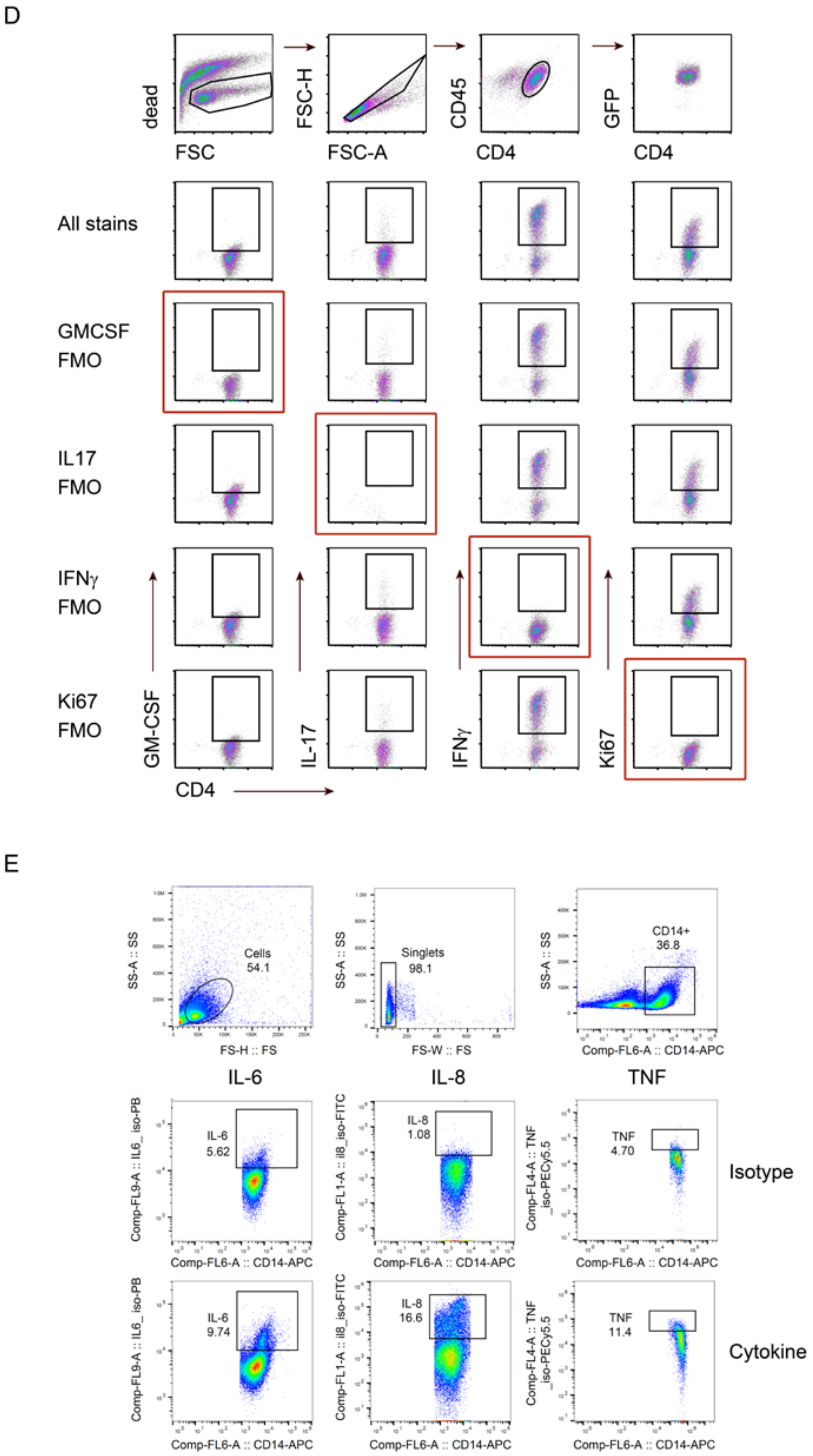

Fig. S2. Gating strategies for flow cytometry. (**A**) Gating strategy for CD4+ T cell activation markers. (**B**) Gating strategy for intracellular cytokine expression and the proliferation marker Ki67. (**C**) Gating strategy for CNS immune cell subpopulations. (**D**) FMO stainings for assessment of rat CD4+ T cell cytokine secretion. (**E**) Gating scheme for assessment of cytokine secretion from human monocytes.

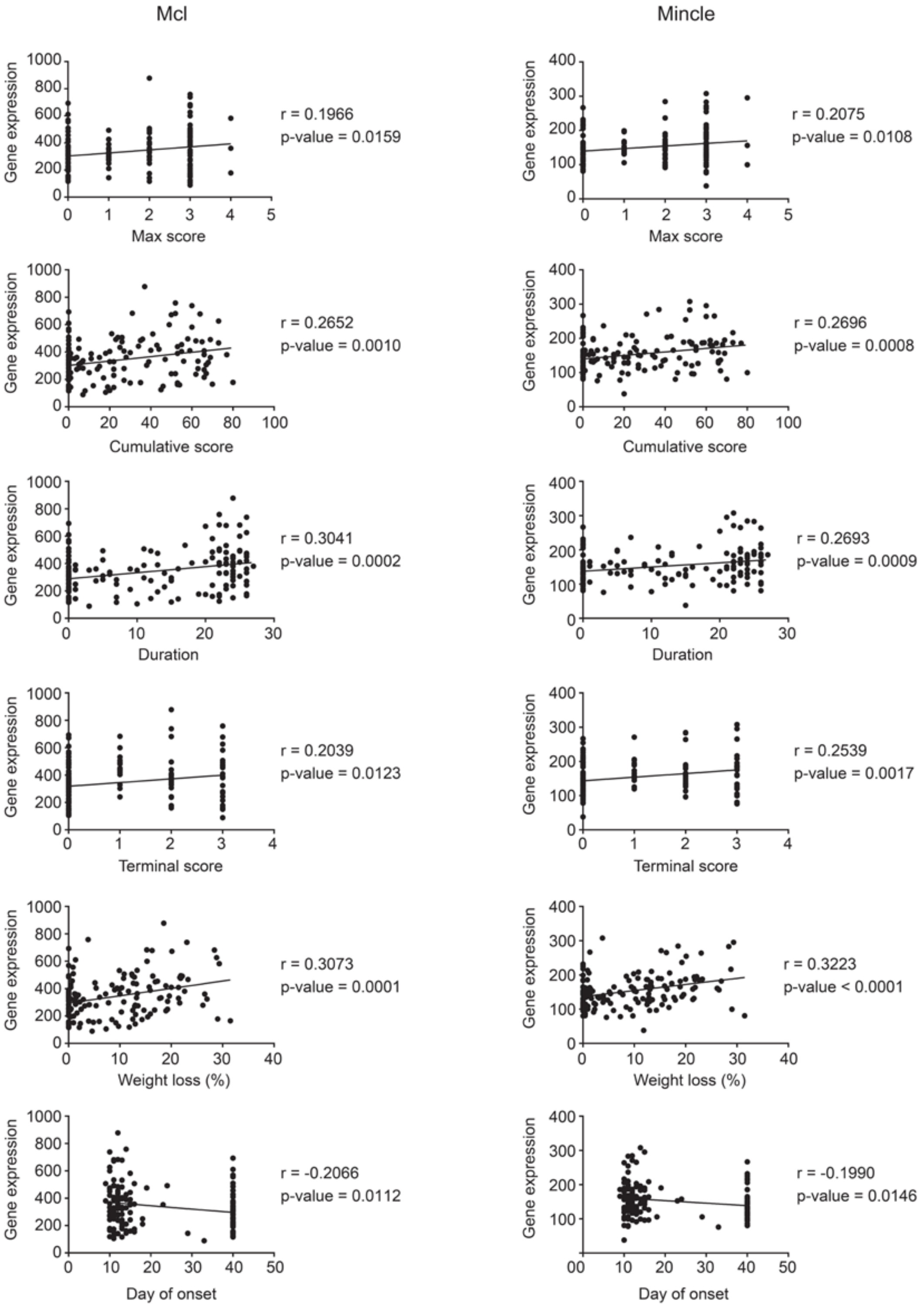

Fig. S3. Correlation between *Mcl/Mincle* expression and clinical EAE parameters. Gene expression from spleens of rats subjected to EAE (*n*=150) was correlated to clinical EAE phenotypes: maximal and cumulative score, disease duration, score at sampling (day 35 p.i.), maximal weight loss (in relation to day 7 p.i.) and day of disease onset. Significant correlations, using Pearson's correlation coefficient (and two-tailed p-value), are depicted in the figure together with lines from linear regression.

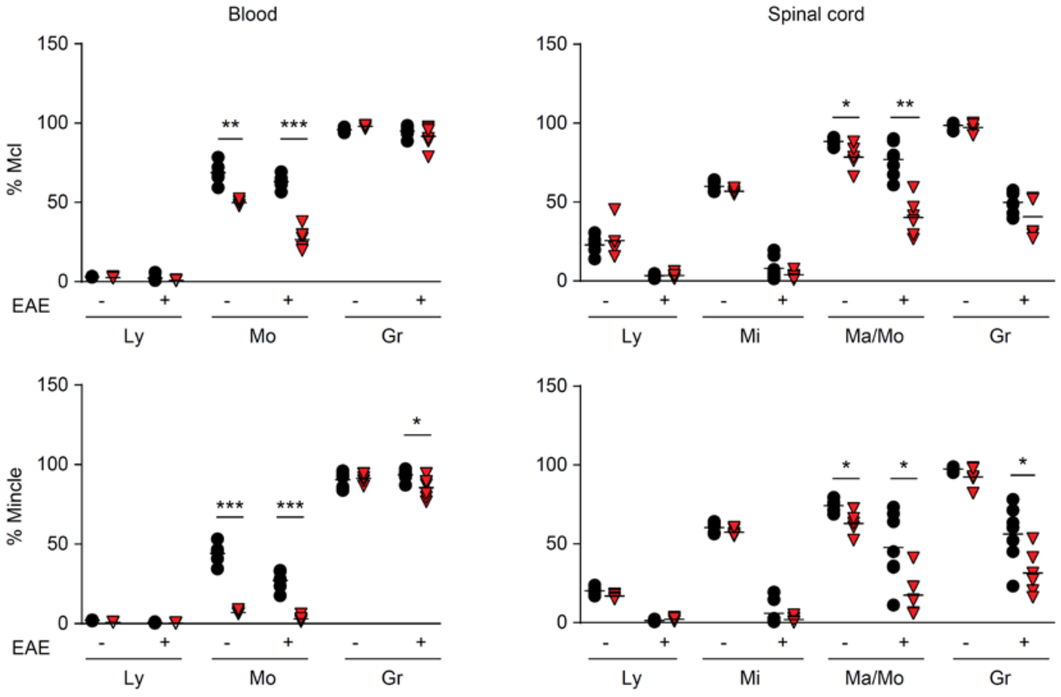

Fig. S4. Reduced frequency of Mcl- and Mincle-expressing cells in CLRc congenic rats. Flow cytometry analysis of cells isolated from peripheral blood and spinal cord of naive DA (n = 5) and CLRc (n = 5) rats and DA (n = 7) and CLRc (n = 6) rats 15 days p.i. The frequencies of Mcl- or Mincle-expressing lymphocytes, monocytes, granulocytes and spinal cord microglia were assessed. All comparisons were done with Mann–Whitney U test. *$P < 0.05$, **$P < 0.01$, ***$P < 0.001$.

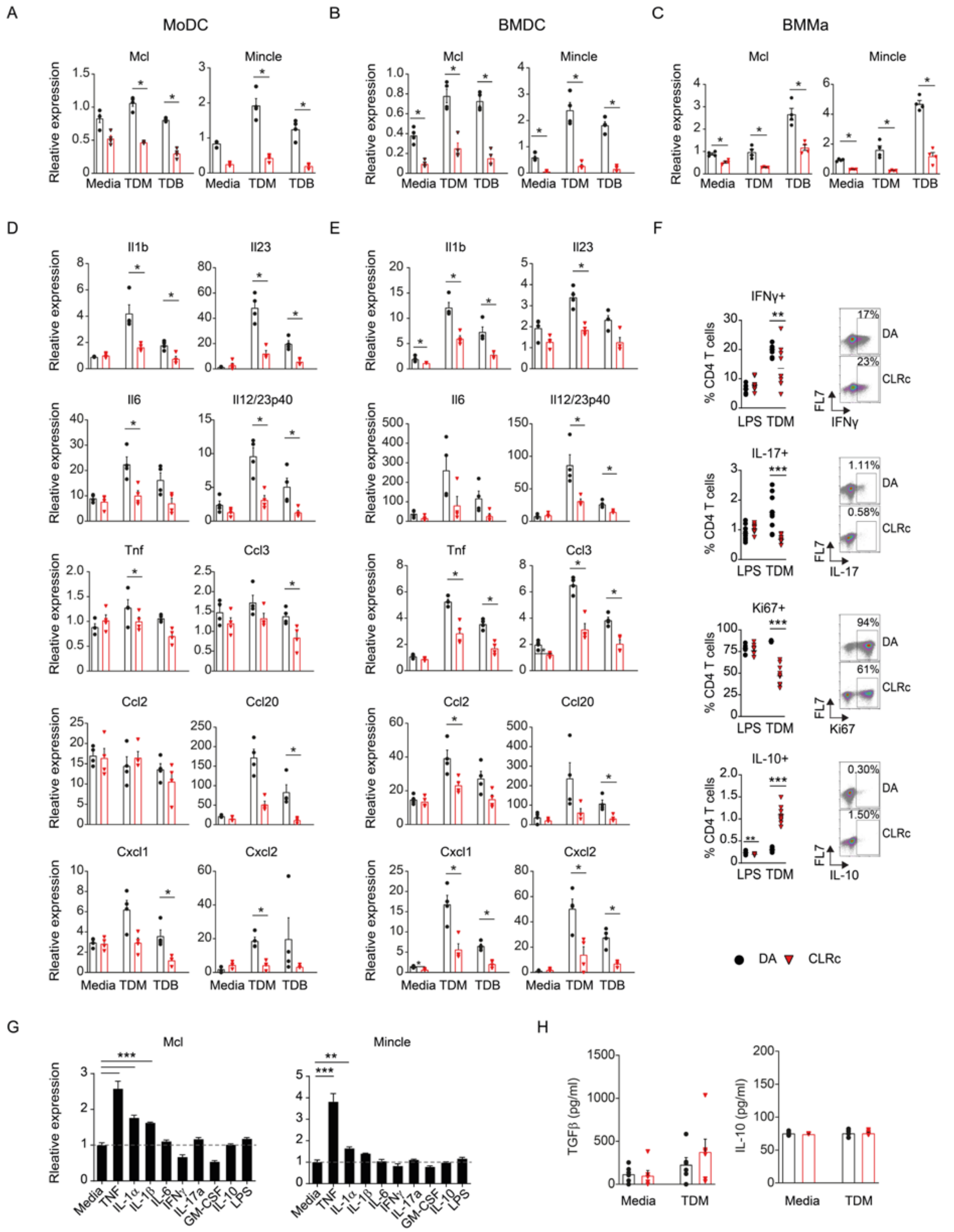

Fig. S5. CLRc bone marrow-derived macrophages and dendritic cells have a reduced response to Mcl/Mincle stimulation. Gene expression level (relative to *Actin*) of *Mcl* and *Mincle* in bone marrow-derived macrophages (BMMa) (**A**), MoDC (**B**) and bone marrow-derived dendritic cells (BMDC) (**C**) from DA (n = 4) and CLRc (n = 4) rats following 18h TDM, TDB and LPS stimulation in vitro (representative of two experiments). qPCR analysis

(relative to *Actin*) of MoDC (**D**) and BMDC (**E**) samples from DA (n = 4) and CLRc (n = 4) rats of transcripts induced in the Mcl and Mincle pathway (representative of two experiments). (**F**) Pooled resting $CD4^+$ cells isolated from lymph nodes of naïve rats were stimulated for 4 days with anti-CD3, anti-CD28 and supernatant from stimulated DA (n=8) or CLRc (n=8) BMDCs. Flow cytometry analysis of $CD4^+$ T cells assessing cytokine production and proliferation (representative of two experiments). (**G**) Mcl and Mincle expression assed by flow cytometry in BMMa from DA rats (n = 4) following cytokine stimulation. (**H**) ELISA for TGFβ and IL-10 from supernatants of DA (n = 6) and CLRc (n = 6) rats from TDM-stimulated MoDC. Data are presented as mean ± SEM. Comparisons were done with Mann–Whitney U test (**A-F** and **H**) and 1-way ANOVA with Dunnett's Multiple Comparison Test (**G**). *P < 0.05, **P < 0.01, ***P < 0.001.

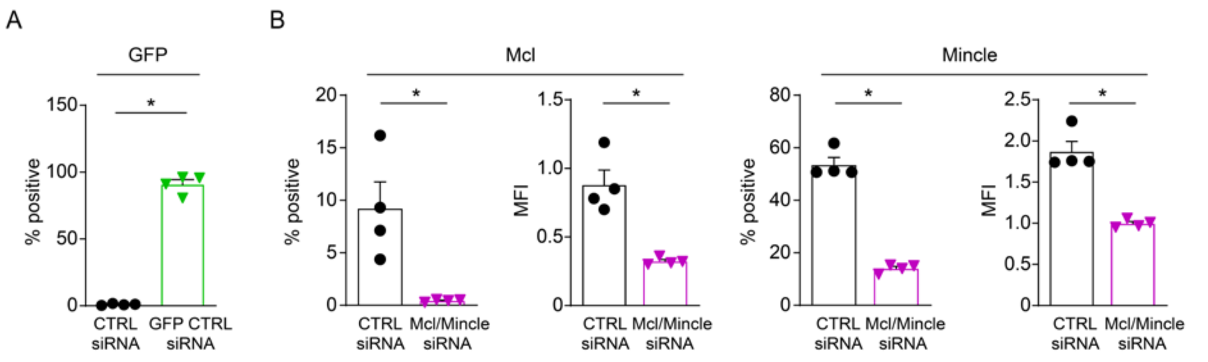

Fig. S6. Evaluation of siRNA silencing of *Mcl* and *Mincle* in vitro. Bone marrow-derived macrophages (BMMa) were treated with *Mcl/Mincle* or scrambled control siRNA. (**A**) Transfection efficiency of BMMa (n=4) transfection with control-siRNA or GFP-control siRNA assessed by flow cytometry. (**B**) Expression of Mcl and Mincle in BMMa assessed by flow cytometry, following treatment with *Mcl/Mincle* siRNA (n=4) or scrambled control siRNA (n=4). Data are presented as mean ± SEM. All comparisons were done with Mann–Whitney U test. *P < 0.05.

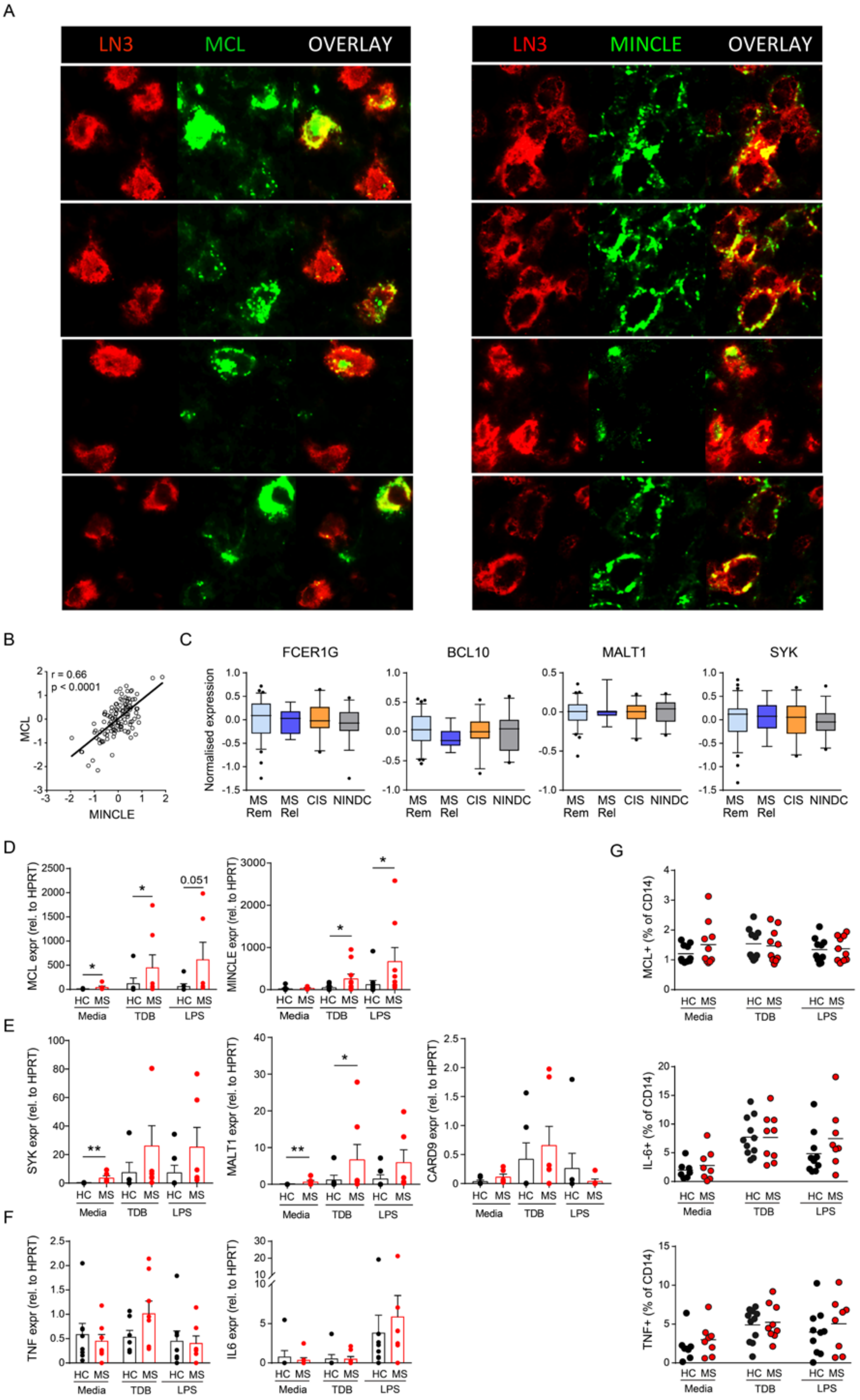
A
LN3
MCL
OVERLAY
LN3
MINCLE
OVERLAY
B
r = 0.66
p < 0.0001
MCL
MINCLE
C
FCER1G
BCL10
MALT1
SYK
Normalised expression
MS Rem
MS Rel
CIS
NINDC
D
MCL expr (rel. to HPRT)
MINCLE expr (rel. to HPRT)
0.051
E
SYK expr (rel. to HPRT)
MALT1 expr (rel. to HPRT)
CARD9 expr (rel. to HPRT)
F
TNF expr (rel. to HPRT)
IL6 expr (rel. to HPRT)
G
MCL+ (% of CD14)
IL-6+ (% of CD14)
TNF+ (% of CD14)
HC
MS
Media
TDB
LPS

Fig. S7. Expression of the MCL/MINCLE signaling pathway in MS patients. (**A**) Additional representative immunofluorescent staining of MS lesions. MCL or MINCLE (green), HLA-DR expression (LN3, red), 40X magnification. (**B**) Expression analysis of PBMC from MS patients and non-inflammatory neurological disease controls (NINDCs) using RNA-sequencing. Expression analysis of PBMC from MS and CIS patients (*n*=115) shows co-expression of *MCL* and *MINCLE* in PBMC. (**C**) Expression of *FCER1G*, *BCL10*, *MALT1* and *SYK* comparing MS patients in remission (n=73), MS patients in relapse (*n*=14), CIS patients (*n*=28) and NINDCs (*n*=36). (**D, E** and **F**) qPCR analysis of *MCL, MINCLE, SYK*, *MALT1, CARD9, TNF* and *IL6* expression in $CD14^+$ enriched fraction from MS patients (*n*=8) and healthy controls (*n*=8) after 24 h stimulation. (**G**) Flow cytometry analysis of MCL, IL-6 and TNF production gated on $CD14^+$ monocytes from MS patients (*n*=9) and healthy controls (*n*=11) after 48 h stimulation. Data are presented as mean ± SEM or box-plots with whiskers representing 5-95 percentile. The following statistical tests were used: Pearson's correlation coefficient with two-tailed p-value (**B**), 1-way ANOVA with Dunnett's Multiple Comparison Test (**C**) and Mann-Whitney U test (**D**, **E**, **F** and **G**). $*P < 0.05$; $**P < 0.01$.

Table S1. Genotyping of the CLRc region on rat chromosome 4.

SNP position (Mb)	159.707	159.746	159.823	159.884	159.906	159.961	160.578
Gene Symbol	*Dcir1*	*Dcar1*	*Dectin-2p*	*Mcl*	*Mincle*	*Vom2r48*	*Pex5*
	Clec4a	*Clec4b2*	*Clec4n*	*Clec4d*	*Clec4e*		
Genotype	DA	**PVG**	**PVG**	**PVG**	**PVG**	**PVG**	DA

Table S2. Correlation between CLR gene expression in spleen and different EAE phenotypes 35 days after EAE induction in (DAxPVG)xDA backcrossed rats (*n*=150).

	EAE (yes/no)	Max EAE score	Onset day	Cumulative EAE score	Duration	Score at day 35	Weight loss
Dcar1	0.05	0.06	0.02	0.05	0.07	0.04	0.05
Dectin-2p	-0.07	-0.03	0.04	-0.05	-0.06	-0.07	-0.09
Mcl	0.20*	0.19*	-0.22**	0.25**	0.28***	0.22**	0.29***
Mincle	0.23**	0.20*	-0.21**	0.26**	0.28***	0.25**	0.31***
Vom2r48	-0.02	0.02	0.06	-0.00	-0.00	0.04	-0.05

Numbers indicate Pearson's correlation coefficient and statistical significance with * $p<0.05$, ** $p<0.01$, *** $p<0.001$.

Table S3. Baseline characteristics of the human brain cohort.

Patient ID	Lesion type	Age (years)	MS type	Gender	Post-mortem delay (h)	Target
MS 1	Chronic active	56	n.a.	n.a.	10:10 h	MCL/MINCLE
MS 2	Chronic active	66	SPMS	F	6 h	MINCLE
MS 3	Active	77	PPMS	M	4:15 h	MCL/MINCLE
MS 4	Active	61	n.a.	n.a.	10:55 h	MCL
MS 5	Active	51	SPMS	M	11:00 h	MCL

n.a. data not available

Table S4. Baseline characteristics of the human RNA-sequencing cohort.

	RRMS relapse (n=14)	RRMS remission (n=73)	PPMS (n=7)	SPMS (n=8)	CIS (n=28)	NINDC (n=36)
Age mean, (SD)	35 (12.4)	40 (12.4)	51 (7.5)	52 (8.4)	36.5 (10.4)	40.9 (12.5)
Female sex, n (%)	9 (64.3)	52 (71.2)	1 (14.3)	5 (62.5)	25 (89.3)	29 (80.5)
EDSS, mean (SD)	2.7 (1.2)	2.0 (1.3)	4.2 (1.1)	4 (0.7)	1.4 (1.0)	n.a.
Years since diagnosis, mean (SD)	1.7 (4.6)	1.7 (4.3)	3 (4.8)	4.9 (9.7)	0.4 (0.7)	n.a.
Immunomodulatory treatment, n (%)	0 (0)	0 (0)	0 (0)	0 (0)	0 (0)	n.a.

n.a. not applicable

Table S5. Baseline characteristics of the human in vitro stimulation cohort.

	MS (n=9)	HC (n=11)	p-value
Age (mean, SD)	57.6 (5.2)	49.9 (13,0)	0.03
Female sex (n, %)	3 (33 %)	4 (36 %)	n.s.
Tysabri treatment (n, %)	9 (100%)	n.a.	n.a.
RRMS, remission (n, %)	9 (100%)	n.a.	n.a.
EDSS (mean, SD)	2.1 (1.4)	n.a.	

n.a. not applicable, n.s. not significant
